# Assessment of the emerging role of AI in diagnosing gliomas using MRI: Systematic review and meta-analysis

**DOI:** 10.1093/noajnl/vdaf162

**Published:** 2025-08-05

**Authors:** Amirah Alsaedi, Walaa Alsharif, Awadia Gareeballah, Sultan Alshoabi, Fahad Alhazmi, Khalid Alshamrani, Lama Alofy, Rahaf Samman, Raneem Al-Bakri, Yara Shukr

**Affiliations:** Department of Diagnostic Radiology, College of Applied Medical Sciences, Taibah University, Madinah, Saudi Arabia; Department of Diagnostic Radiology, College of Applied Medical Sciences, Taibah University, Madinah, Saudi Arabia; Department of Diagnostic Radiology, College of Applied Medical Sciences, Taibah University, Madinah, Saudi Arabia; Department of Diagnostic Radiology, College of Applied Medical Sciences, Taibah University, Madinah, Saudi Arabia; Department of Diagnostic Radiology, College of Applied Medical Sciences, Taibah University, Madinah, Saudi Arabia; Ministry of the National Guard - Health Affairs, Jeddah, Saudi Arabia; King Abdullah International Medical Research Center, Jeddah, Saudi Arabia; College of Applied Medical Sciences, King Saud bin Abdulaziz University for Health Sciences, Jeddah, Saudi Arabia; Department of Diagnostic Radiology, College of Applied Medical Sciences, Taibah University, Madinah, Saudi Arabia; Department of Diagnostic Radiology, College of Applied Medical Sciences, Taibah University, Madinah, Saudi Arabia; Department of Diagnostic Radiology, College of Applied Medical Sciences, Taibah University, Madinah, Saudi Arabia; Department of Diagnostic Radiology, College of Applied Medical Sciences, Taibah University, Madinah, Saudi Arabia

**Keywords:** AI, glioma, grading, MRI

## Abstract

Despite the emerging role of artificial intelligence (AI) in glioma grading, its clinical adoption remains in its early stages. This meta-analysis aims to assess the role of AI in differentiating glioma grades using magnetic resonance imaging (MRI). Twenty-five studies matched the inclusion criteria and were included after systematic searches through “PubMed” electronic database. The quality of the included studies was assessed utilizing Quality Assessment of Diagnostic Accuracy Studies-2 (QUADAS-2). A bivariate random‐effects model was employed to estimate the pooled effect of the sensitivity and specificity, followed by an estimation of the summary receiver operating characteristic (SROC) curve. The overall results suggest relatively high sensitivity and specificity among the assessed AI methods for discriminating glioma grades. Convolutional Neural Networks (CNN) demonstrated the highest diagnostic accuracy, with a sensitivity of 93% (95% CI: 88%-97%) and specificity of 92% (95% CI: 90%-94%). This meta-analysis highlights the potential role of AI models based on MRI in supporting clinicians in glioma grading.

**Key Points**

- This study highlights the promise of AI-based methods, particularly CNNs, for glioma grading.
- While these tools hold significant potential to enhance diagnostic workflows, their integration into clinical practice requires rigorous validation, standardization, and efforts to minimize the impact of selective reporting of high-performing models.
- By addressing these challenges, AI can become a transformative tool in the management of gliomas, improving outcomes for patients worldwide.

**Importance of the Study**

This work’s findings synthesize evidence from multiple studies to provide critical benchmarks for AI performance. The study also demonstrated how AI enhances glioma grading and emphasizes AI’s potential role in reducing invasive diagnostics. Overall, this research highlights the importance of AI in glioma grading, improving patient outcomes, and shaping the future of healthcare delivery.

Gliomas are the most common type of central nervous system (CNS) tumors that arise from glial cells.^1^ Currently, the World Health Organization (WHO) classifies gliomas into four grades: grades I and II, which are considered low-grade gliomas (LGG), grades III and IV, which are considered high-grade gliomas (HGG).^2^ Accurately defining glioma grade directly impacts the treatment plan and prognosis. Magnetic resonance imaging (MRI) plays a crucial role in this process.^3^ In previous years, clinicians could only obtain basic tumor information by analyzing conventional MR images (eg, enhanced T1-weighted [T1-W] and Fluid-Attenuated Inversion Recovery [FLAIR] images).^3^ However, advanced MRI methods, including diffusion-weighted imaging (DWI), perfusion imaging, and magnetic resonance spectroscopy (MRS), have significantly improved the differentiation of tumor grades.^3,4^ Despite these advancements, limitations remain, as human diagnosis can be time-consuming and prone to subjective interpretation, leading to variability in diagnosis.^5^

Recent studies have highlighted the potential role of artificial intelligence (AI) in enhancing glioma grading by analyzing complex patterns in MRI images and capturing lesion heterogeneity that is imperceptible to the human eye.^5,6^ A study by Cho et al. utilized a random forest classifier based on routine brain MRI and reported an AUC of 0.92 for differentiating glioma grades.^7^ Another study by Citak-Er et al. developed a machine learning (ML) model for glioma grading using a support vector machines (SVM) algorithm based on multi-parametric MRI (mp-MRI) features. The model demonstrated high diagnostic performance, achieving an accuracy of 93.0%, a sensitivity of 96.4%, and a specificity of 86.7%.^8^

Despite the emerging role of AI in glioma grading, its clinical adoption remains in its early stages. This meta-analysis aims to evaluate the effectiveness of AI in differentiating glioma grades using MRI. By synthesizing and analyzing existing evidence, this study seeks to establish a comprehensive understanding of AI’s diagnostic performance, including its accuracy, sensitivity, and specificity. The findings of this meta-analysis could provide critical insights into the potential of AI to overcome the limitations of traditional diagnostic methods, such as time consumption and subjective variability. Ultimately, this research could pave the way for the clinical integration of AI, enabling more precise, efficient, and standardized glioma grading, which is essential for optimizing treatment planning and improving patient outcomes.

## Materials and Methods

### Literature Search, Selection, and Data Extraction

Our study followed the Preferred Reporting Items for Systematic reviews and Meta-Analyses (PRISMA) guidelines, addressing the research question of “What is the emerging role of artificial intelligence in the diagnosis of adult gliomas using magnetic resonance imaging?.”^9^ We identified search terms based on the Population/Intervention/Comparator/Outcomes (PICO) framework, linking terms with Boolean operators (“OR” within each PICO category and “AND” between PICO categories).

The search terms were categorized as follows:

- **Concept 1 (P):** glioma OR neuroglia OR oma
- **Concept 2 (I):** artificial intelligence OR Machine learning OR deep learning OR magnetic resonance imaging OR MRI
- **Concept 3 (O):** diagnosis OR grading OR differentiate

**Figure 1.**
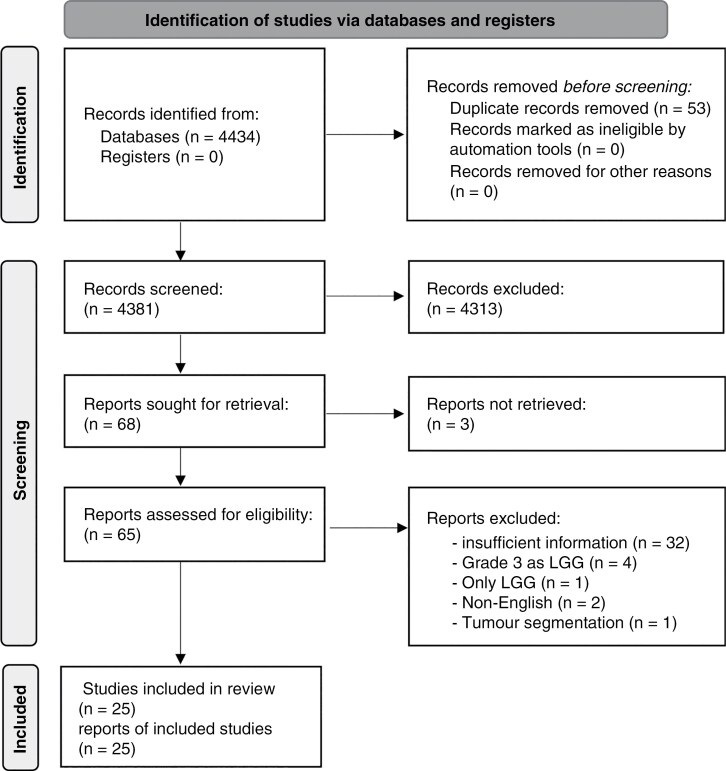
Preferred Reporting Items of Systematic Reviews and Meta-Analyses-PRISMA flow chart for the study selection process.

To broaden the search, we also conducted an iteration without including the third concept. Consequently, the final search strategy combined the terms from steps 1, 2, and 3 as follows: [(glioma OR neuroglia OR oma) AND (artificial intelligence OR machine learning OR deep learning OR radiomics OR magnetic resonance imaging OR MRI) AND (diagnosis OR grading OR differentiation)]. The systematic search was conducted in 2022 using the PubMed electronic database, filtering results from 2010 to 2022 to identify relevant articles that met the predefined search terms. As illustrated in the PRISMA flow diagram (Figure 1), this process initially identified 4,434 articles, which were reduced to 4,385 after the removal of duplicates.

The inclusion criteria for the review were as follows:

(a) Original research articles;
(b) Studies involving patients with histopathologically confirmed WHO grade gliomas, including both lower-grade and high-grade gliomas;
(c) Research focusing on preoperative glioma grading using MRI and AI, ML, or DL models as primary diagnostic tools;
(d) Studies with full-text availability and sufficient information.

A total of 4,317 articles were excluded for not meeting these criteria. Reasons for exclusion included:

- Studies not utilizing AI for glioma grading (eg, Abrigo et al., 2018)^4^;
- Non-glioma grading studies (eg, Karri et al., 2022)^10^;
- Articles focused on IDH mutations (eg, Cao et al., 2021)^11^;
- Non-original research articles (eg, Sohn et al., 2020)^12^;
- Studies not involving MRI (eg, Peng et al., 2021)^13^;
- Research applying reference standards or index tests on pediatric patients (eg, Zhang et al., 2021)^14^ or non-human subjects (eg, Young et al., 2011)^15^;
- Non-grading studies focused on diagnosis, treatment, or management (eg, Wei et al., 2022)^16^;
- Studies on postoperative or recurrent gliomas (eg, Young et al., 2012)^17^;
- Non-English studies (eg, Mao et al., 2018).^18^

Additionally, after a detailed review, studies lacking validation^19^ or sufficient data^20^ (eg, sensitivity, specificity, or confusion matrix) were excluded. Ultimately, 25 studies met the inclusion criteria and were deemed eligible for the review, listed in Table 1.^7,21–44^

**Table 1.** Information of the Included Studies

AI model	Feature extraction	Feature reduction	ROI	MRI method	Testing sample	Training Sample	Validation	Institutional/public	Code availability	WHO-grading system	Author (years)	No.
SVM	Histogram	PCA, ICA, PCC	Automatic from the anatomical MR images	T2, T1,T1-CE, DSC	“leave-m-out scheme”	101 (63HGGs, 38LGGs)	k-fold cross-validation scheme with 10 training and test sets to validate the SVM models	Institutional	No	WHO-2007	^21^	1
SVM	SIDR, *SIC*, fALFF, ReHo	Not provided	The region of interest (ROI) was adjusted semi-automatic to cover the whole tumor in both 3D T1-weighted images and RS-fMRI.	T1, RS-fMRI	15 (7 HGGs, 8 LGGs)	20 (10 HGGs, 10 LGGs)	train-test split	Institutional	No	WHO-2007	^32^	2
SVM RF	The peak integral values of four metabolites (creatine (two resonant peaks), choline and NAA)	Not provided	The MR spectrum was acquired at different areas on the tumor	Multivoxel MRS	fivefold cross-validation scheme	28 (12 HHGs, 16 LGGs)	fivefold cross-validation scheme	Institutional	No	WHO-2007	^38^	3
SVM LR RF	Histogram, shape, GLCM, GLRLM, GLSZM, NGTDM, GLDM	LASSO and mRMR	semi-automatic/ TC = (NCR, ET and NET) on T1GD semi-automatic / PED on FLAIR	T1,T1-CE,T2 and FLAIR	BraTS 2019 dataset	BraTS 2017 dataset (420 with SMOTE)	five-fold cross-validation	Public (BraTs2017 & BraTS2019)	Yes (https://github.com/chengjianhong/glioma_grading.git)	WHO-2007 and 2016	^39^,	4
SVM	GLCM, GLGCM, Histogram	SVM-RFE	Manually delineated on the T1- CE image and avoided the inclusion of obvious cysts regions, with the T1WI, T2WI, and DWI	T1,T1-CE,T2, Diffusion, Structure, 3D pCASL	10-fold cross- validation	153(111HGGs, 42 LGGs), 222 with SMOTE	10-fold cross-validation	Institutional	No	WHO-2007 and 2016	^40^	5
SVM	Histogram, TA	SVM-RFE	Manually drawn around the whole FLAIR abnormality	DKI, FLAIR	LOOCV	37 (18 HGGs, 19 LGGs)	nested leave-one-out cross validation (LOOCV)	Institutional	No	WHO-2016	^41^	6
SVM LR RF	shape-based, Histogram, GLCM, ISZ	mRMR	Semi-automatic, ROI1 (NCR + NET), ROI2 (NCR + NET + ET), ROI3 (NCR + NET + ET + PTE)	T1, T1-CE, T2 and FLAIR	five-fold cross validation	BraTS 2017 dataset 285 (210 HGGs, 75 LGGGs)	five-fold cross-validation	Public (BraTs2017)	Yes (https://pmc.ncbi.nlm.nih.gov/articles/instance/6252243/bin/peerj-06-5982-s003.zip)	WHO-2007	^7^	7
SVM	Histogram, GLCM, GLRLM	SVM-RFE	A semi-automated segmentation technique, based on T1W-C and DTI, was used for tumor core delineation in all available para- metric maps	conventional (T1, T1-CE, T2 and FLAIR) and advanced techniques DTI, Perfusion, MRS	LOOCV	40 (20 HGGs, 20 LGGs)	leave-one-out cross-validation (LOOCV)	Institutional	No	WHO-2007/2016	^42^	8
SVM	1H-MRS metabolic features	mRMR	A neuroradiologist delineated the tumor area on T2-weighted images to include the largest tumor region	Multivoxel MRS	38 (19 HGGs, 19 LGGs)	74 (31 HGGs, 43 LGGs)	train-test split	Institutional	Yes (https://pmc.ncbi.nlm.nih.gov/articles/instance/6487359/bin/mmc1.docx).	WHO-2016	^43^	9
SVM	Deep features, texture features, and shape/morphological features	Not provided	Manually on the B0 images, ROI1 (denotes all abnormal signals on the B0 image, including the contrast-en- hancing, peritumoral edema, cyst, and necrotic regions), while ROI2 (just contains the solid part of the tumor, excluding necro- sis, cyst, and peritumoral edema)	T1-IR, T2, FLAIR,T1-CE and MD and FA maps from DTI	LOOCV	108 (65 HGGs, 43 LGGs)	leave-one-out cross-validation (LOOCV)	Institutional	No	WHO-2016	^44^	10
SVM-RBF LASSO-LR RF SVM-L	First-order, GLCM, GLSZM, GLRLM, NGTDM, GLDM.	select the important radiomic features and train each model using 5-fold cross- validation	semi-automatic segmentation of the solid portion of the tumor avoiding any cysts, calcifications, edema, hemorrhage, necrosis, and large vessels using 3 conventional MR sequences (T2-FLAIR, T2WI, and T2*WI)	ADC-map from DWI and CBF-map from pCASL	16 (10 HGGs, 6 LGGs)	48 with SMOTE (24 HGGs, 24 LGGs)	Of the sample 70% training, and 30% testing	Institutional	No	WHO-2007/2016	^22^	11
SVM	First-order, GLCM, GLSZM, GLRLM, NGTDM, GLDM. All derived from unfiltered images, from wavelet filtered images and from LoG filtered images	ANOVA F-test, mRMR, RFE, a wrapper-based approach	Manually segmented guided by T2-FLAIR hyperintensity, whole for LGG, while for the HGG T2-FLAIR hyperintense region beyond the contrast-enhancing tumor core.	T1-CE, T2-FLAIR, ADC-map from DWI	LOPO) cross- validation	74 (42 HGGs, 32 LGGs)	Leave-one-out cross-validation	Institutional (available on request)	Yes (The radiomic feature extraction was performed using freely available Pyradiomics software (http://www.pyradiomics.io/ pyradiomics.html). All standardization, model fitting, and assessment were performed using Scikit-Learn (https://scikit-learn.org/stable))	WHO-2016	^23^	12
SVM	HOG	“local to whole” approach	In this study, they designed an automatic pipeline to analyze the image features without manual or semi-manual ROI delineation.	T1, T2, and T2-FLAIR	10-fold cross-validation	Public TCIA datasets 134 (76 HGGs, 58 LGGs)	stratified 10-fold cross-validation	public TCIA datasets	No (available on request)	WHO-2007/2016	^24^	13
SVM	Histogram	SVM-PCA, SVM-RFE, SVM-LASSO	Manually drawn on CE-T1WI including the solid portions, hemorrhage, cystic change, and necrosis were selected as VOIs and peritumoral edema was excluded.	DKI	LOOCV	161 (100 HGGs, 61 LGGs)	nested leave-one-out cross validation (LOOCV)	Institutional (available on request)	No (available on request)	WHO-2016	^25^	14
SVM LR RF	Deep learning features and radiomics features (First-order, GLCM, GLRLM, GLSZM, GLDM, and NGTDM, shape features)	Spearman correlation test and RF-RFE	The gross tumor volumes (GTVs) were manually delineated on the axial MPR images by an oncologist using the 3D Slicer software.	Multiplanar CE-T1W MPR imaging	50 (25 HGG, 25 LGG), 20 of the from the public TCIA datasets (8 HGGs, 12 LGGs)	101 (43 HGGs, 58 LGGs)	five-fold cross-validation was performed 500 times	Both	Yes (https://github.com/ljljlj02/deep-learning-radiomics-feature-extraction)	WHO-2016	^26^	15
LR	TA and histogram	Spearman correlation	A two-dimensional ROI enclosing the largest cross-sectional area of the tumor was manually delineated on FLAIR, ADC, and post-contrast T1 images by three readers where Cystic, necrotic, or hemorrhagic areas were included	T1-CE, FLAIR and DWI, ADC-map from DWI	Not provided	94 (80 HGGs, 14 LGGs)	Not provided	Institutional	No	WHO-2016	^27^	16
LR	Histogram features, shape and size based features,texture features, wavelet features	Correlation analysis	Semi-automatic method based on the signal intensity threshold in tumor lesions was implemented to depict the tumor area, tumor areas were mainly defined as areas with hyperintensity on T2-FLAIR and T1C images with contrast enhancement. With the aid of DWI, necrotic and cystic areas were avoided.	T2-FLAIR, DWI, DKI (ADC, Dmean, FA and MK maps)	the obtained models were validated within 1000 × bootstrapping	139 (70 HGGs, 69 LGGs)	the obtained models were validated within 1000 × bootstrapping	Institutional	No	WHO-2016	^28^	17
LR	Clinical imaging diagnostic features and hand crafted radiomics (Histogram, form factor, texture, GLCM, and Haralick features, RLM, GLSZM).	Univariate statistical tests and LASSO	Delineated around the whole tumor manually on each sequence by selecting the largest lesion in the presence of multiple lesions.	T1-WI, T1-CE, T2-WI, T2-FLAIR	Not provided	59 (46 HGGs, 13 LGGs)	Not provided	Institutional	No	WHO-2016	^29^,	18
LR	Histogram, Haralick feature, morphology, GLCM, RLM, GLZSM	Spearman correlation analysis and LASSO	Semiautomatic method with an interactive level-set volume of interest via threshold-based and edge-based algorithms, outlined ROI layer by layer on all levels of the displayed tumor in the enhanced T1WI sequences.	T1WI, T2WI, and T1-CE	34 (24 HGGs, 10 LGGs)	80 (55 HGGs, 25 LGGs)	train-test split in a ratio of 7:3	Institutional (available on request)	No (available on request)	WHO-2016	^30^,	19
CNN	Deep CNN features		Focusing feature learning on the tumor areas instead of the whole brain	T1, T1-CE, T2, FLAIR	86 (63 HGGs,23 LGGs)	MICCAI dataset GAN-augmented data are used (126 HGGs, 45 LGGs)	hold-out validation where the dataset was partitioned into 3 subsets: training (60%), validation (10%), and testing (30%)	Public MICCAI dataset	KERAS library [Keras. version 2.2.4, https://github.com/fchollet/keras. Accessed 03 Oct 2018.] with TensorFlow [TensorFlow: Large-Scale Machine Learning on Heterogeneous Systems. Version 1.14.0, https://www.tensorflow.org/. Accessed 19 June 2019.] back- end was used	WHO-2007	^31^	20
CNN	CNN architecture		user-guided-largest tumor, outlined in the MRI slices with the largest tumor cross-section and centered on the corresponding tissues	T2W\FLAIR	five-fold cross validation	104 (54 HGGs,50 LGGs) data augmentation techniques was used	nested five-fold cross validation	Institutional (available on request)	No, The custom convolutional neural network (available on request), AlexNet (A. Krizhevsky, I. Sutskever, G. E. Hinton, ImageNet classification with deep convolutional neural networks, Commun. ACM, 60 (2017), 84–90.), GoogleNet (C. Szegedy, Wei Liu, Yangqing Jia, P. Sermanet, S. Reed, D. Anguelov, et al., Going deeper with convolutions, Proceedings of the 28th IEEE Conference on Computer Vision and Pattern Recognition, USA, 2015.), SqueezeNet (F. N. Iandola, S. Han, M. W. Moskewicz, K. Ashraf, W. J. Dally, K. Keutzer, SqueezeNet: AlexNet-level accuracy with 50 × fewer parameters and < 0.5mb model size, preprint, arXiv:1602.07360.)	WHO-2007/2016	^33^	21
CNN	architecture of the proposed HOMIF framework		There are masks available for the tumors of BraTs2017.	T1, T1-CE, T2, and T2-FLAIR	Five-fold cross validation	BraTS 2017 dataset (285, 210 HGG,75 LGG). Of them 80% training and 20% validation subset	Five-fold cross validation	Public (BraTs2017)	No	WHO-2007	^34^	22
RF	lesion size on T2, percentage of non-enhancing base on T1 and T2, location of the tumor was based on the lobe of the brain that contained the geographic base on T2 and FLAIR, non-enhancing border of the tumor base on T1 and T2, minimal ADC value in the tumor	Not proided	Not provided	T1, T1-CE, T2-W, FLAIR and DWI	Five-fold cross validation	381 patients (324 HGGs, 57 LGGs) where SMOTE method was applied to generate synthetic samples to help balance the training set.	Five-fold cross validation	Institutional	No	WHO-2016	^35^	23
RF	first-order, GLCM, GLRLM, GLSZM, GLDM, NGTDM, and shape Feature	Pearson correlation analysis, RF classifier	Semi-automatically segment the whole tumor enhancement were manually drawn on the T1 CE and FLAIR slice by slice	T1-CE and FLAIR	Five-fold cross validation	T1-CE for 36 (17 HGGs,19 LGGs) and FLAIR for 33 (16 HHGs, 17 LGGs)	Five-fold cross validation	Institutional (available on request)	No	WHO-2007/2016	^36^	24
RF	First-order, GLCM, GLRLM, GLSZM, shape Features	PCA	Two neuroradiologists (5 years of experience) drew the region of interest (ROI) around the tumor boundary on the T1-CE images.	T1-CE	93	276 increased to 318 with SMOTE	grid search with cross-validation was applied	Institutional (available on request)	No	WHO-2007/2016	^37^	25

^*^ **Abbreviations:** Three-Dimensional Pseudo-continuous Arterial Spin Labeling (3D pcASL), Two-Dimensional(2D), Apparent Diffusion Coefficient (ADC), Area Under The Receiver Operating Characteristic Curve (AUC), Contrast Enhancement (CE), Convolutional Neural Network (CNN), Cancer Imaging Archive (TCIA) public repository, Diffusion Weighted Image (DWI), Diffusional Kurtosis Imaging (DKI), mean diffusion coefficient (Dmean), Enhancing Part Of The Tumor Core (ET), Fluid-Attenuated Inversion Recovery (FLAIR), Fractional Amplitude Of Low-Frequency Fluctuation (fALFF), Fractional Anisotropy (FA), Generative Adversarial Networks (GANs), Glioblastoma (GBM), Gray Level Co-Occurrence Matrix (GLCM), Gray Level Dependence Matrix (GLDM), Gray Level Run Length Matrix (GLRLM), Gray Level Size Zone Matrix (GLSZM), Gray-Level Gradient Co- Occurrence Matrix (GLGCM), High-Grade Glioma (HGG), hierarchical-order multimodal interaction fusion network (HOMIF), Histogram of Oriented Gradients (HOG) algorithm, Intensity Size-Zone [ISZ], independent component analysis (ICA), Laplacian Of Gaussian (Log), Least Absolute Shrinkage And Selection Operator (LASSO), Low-Grade Glioma (LGG), leave-one-patient-out (LOPO) cross- validation, Magnetic Resonance Spectroscopy (MRS), Mean Diffusivity (MD), Minimum Redundancy Maximum Relevance (mRMR), multiplanar reconstruction (MPR), mean kurtosis (MK), n-acetyl aspartate (NAA), Neighboring Gray Tone Difference Matrix (NGTDM), Nested Leave-One-Out Cross Validation (LOOCV), Non-enhancing Part Of The Tumor Core (NET), Peritumoral Region (PTR), Random Forest (RF), Principal Component Analysis (PCA), Pearson’s correlation coefficients (PCC), Region Of Interest (ROI), Regional Homogeneity (Reho), Resting State Blood Oxygenation Level-Dependent Functional Magnetic Resonance Imaging (RS-fMRI), Run-Length Matrix (RLM), Signal Intensity Correlation (*SIC*), Signal Intensity Difference Ratio (SIDR), Synthetic Minority Over-sampling Technique (SMOTE), Support Vector Machine (SVM) With The Radial Basis Function Kernel (SVM-RBF), SVM With The Linear Kernel (SVM-L), SVM-Based Recursive Feature Elimination (SVM-RFE), T1 Inversion Recovery (IR), T1-Weighted Image(T1WI), T2-Weighted Image(T2WI), Texture Analysis (TA), The Necrotic (Fluid-Filled) (NCR), The Peritumoral Edema (PED), Tumor Core (TC), T2-weighted fluid-attenuated inversion recovery (T2- FLAIR), Fluid-attenuated inversion recovery (FLAIR), Visually Accessible Rembrandt Images (VARSARI).

After identifying the appropriate group of studies, relevant performance assessment indices were extracted, including sensitivity, specificity, true positives (TP), false positives (FP), false negatives (FN), and true negatives (TN). The Quality Assessment of Diagnostic Accuracy Studies-2 (QUADAS-2)^45^ tool was employed by two independent reviewers to provide a transparent evaluation of the risk of bias and the applicability of the included studies.

### Statistical Analyses

The diagnostic performance (sensitivity and specificity) of the AI methods used to differentiate between HGGs and LGGs was evaluated. Some studies reported multiple diagnostic outcomes from the same sample, achieved through either different MRI techniques or variations in the image post-processing pipeline. To prevent overestimating the study weight, outcomes from the same study were treated as originating from separate studies. These studies are listed in Table 1A in the appendices.

**Figure 2.**
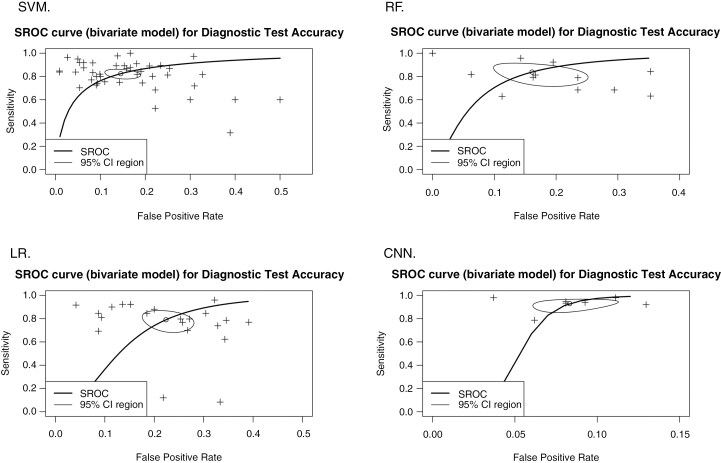
SROC plots of the summary curve of the sensitivity and the specificity (open circle) and its 95% CI region of the assessed AI methods (SVM, RF, LR, and CNN) to differentiate between LGGs and HGGs. SVM, support vector machine; RF, random forest; LR, logistic regression; and CNN, convolutional neural network.

**Figure 3.**
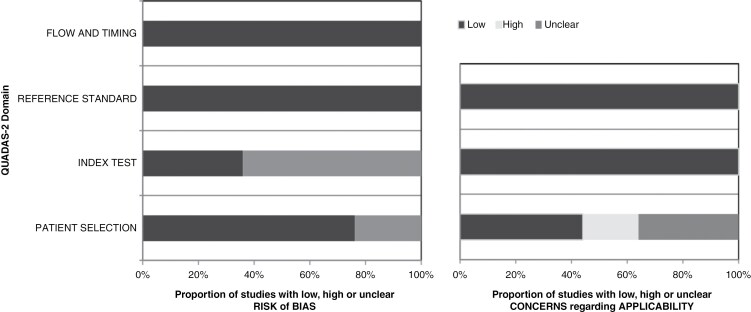
The Quality Assessment of Diagnostic Accuracy Studies-2 (QUADAS-2) results for the included studies.

The analysis strategy employed followed the approach outlined by Shim SR et al,^46^ where a bivariate random‐effects model was employed to estimate the pooled sensitivity and specificity. This was followed by the construction of a summary receiver operating characteristic (SROC) curve. Study heterogeneity was assessed quantitatively using the *Q*-test and *I*^2^ statistics. A *Q*-test *P*-value < 0.05 and/or an *I*² value > 50% were considered evidence of substantial heterogeneity. The forest plots of the pooled sensitivity, specificity, and the funnel plots from the SVM, RF, LR, and CNN models are shown in Figures 1A-12A in the appendices.

Potential publication bias was evaluated using the Egger test, with a p-value < 0.01 indicating significant asymmetry and the presence of publication bias. meta-regression was used to explore the heterogeneity. Diagnostic odds ratio (DOR) was used for the assessment of the publication bias and the heterogeneity since it depends significantly on sensitivity and specificity. All analyses were performed using the “mada” and “meta” packages in R Statistical Software (Version 1.4.1103, R Foundation for Statistical Computing, Vienna, Austria).

## Results

The overall results suggest relatively high sensitivity and specificity among the assessed AI methods for discriminating glioma grades. Convolutional Neural Networks (CNN) demonstrated the highest diagnostic accuracy, with a sensitivity of 93% (95% CI: 88%-97%) and specificity of 92% (95% CI: 90%-94%), indicating excellent performance and minimal heterogeneity in specificity.

Random Forest (RF) and Support Vector Machines (SVM) exhibited comparable diagnostic performance, with sensitivity and specificity of approximately 87%. Logistic regression (LR), on the other hand, showed lower sensitivity (80%, 95% CI: 70%-87%) and specificity (79%, 95% CI: 74%-83%) compared to other methods. Heterogeneity varied across methods, being moderate to high, particularly for sensitivity in RF (*I*² = 82%) and LR (*I*² = 79%).

Publication bias was detected for RF (Egger test *P* = 0.008) and CNN (Egger test *P* = 0.009), suggesting caution in interpreting the findings for these methods. A summary of the diagnostic performance, including sensitivity, specificity, and publication bias, is presented in Table 2. The SROC plots are shown in Figure 2.

**Table 2.** Diagnostic Performance of the Assessed AI Methods in Discrimination Between Glioma Grades

AI method	Number of studies included	Sensitivity (95% CI)	Heterogeneity		Specificity (95% CI)	Heterogeneity		Egger test to evaluate publication bias
			*I* ^2^	*P*-value		*I* ^2^	*P*-value	*P*-value
SVM	44	84% (80%, 89%)	69%	<.01	87% (84%, 90%)	75%	<.01	.29
RF	15	87% (77%, 93%)	82%	<.01	87% (79%, 92%)	53%	<.01	.008
LR	22	80% (70%, 87%)	79%	<.01	79% (74%, 83%)	72%	<.01	.37
CNN	7	93% (88%, 97%)	69%	<.01	92% (90%, 94%)	0%	.57	.009

Table 2A in the appendices summarizes the results for each AI model and moderator. For the Support Vector Machine (SVM) model, MRI image weighting explained the largest proportion of heterogeneity (*R*² = 80.5%, *P* < .001), followed by region-of-interest (ROI) selection (*R*² = 56%, *P* < .001) and feature extraction methods (*R*² = 50%, *P* = .002). These moderators significantly reduced residual heterogeneity (QE, *P* < .01), indicating their substantial influence on performance variability.

For the logistic regression (LR) model, feature reduction (*R*² = 61%, *P* = .003), ROI selection (*R*² = 60%, *P* = .003), and MRI image weighting (*R*² = 63%, *P* = .004) significantly contributed to explaining heterogeneity. Feature extraction methods did not account for heterogeneity in this model (*R*² = 0%, *P* = .520).

For the Random Forest (RF) model, feature reduction and ROI selection each accounted for 100% of heterogeneity (*P* < .001), effectively eliminating residual between-study variance (QE *P* > .5). MRI image weighting also explained a high proportion of heterogeneity (*R*² = 90.7%, *P* < .001), though some residual heterogeneity remained (*I*² = 39.7%).

We did not perform meta-regression for the Convolutional Neural Network (CNN) model, as it was evaluated in only seven studies, below the commonly recommended threshold of ten studies for reliable meta-regression analysis.^47^ Still, the heterogeneity can be noticed by glancing at the forest plots of the pooled sensitivity, specificity (Figures 10A and 11A) in the Supplementary Material.

### QUADAS-2 Assessment

The studies’ risk of bias and applicability are presented in the summary graph of the QUADAS-2 assessment in Figure 3. There are four domains in the risk of bias graph: the patient selection domain, where 24% of the included studies did not explain whether inappropriate exclusions were avoided or not (unclear); the index test domain introduced 64% bias as the majority of studies, which the authors did not mention if the neuroradiologists were blinded to histopathological findings (unclear); the reference standard domain and flow and time domain which both showed a low risk of bias. Regarding the applicability graph, there were three domains: the patient selection domain, where 36% of the studies did not provide information about patients’ age (unclear), 20% (high) as four studies included pediatric and adult patients, one study included postoperative glioma patients; the index test and the reference standard, both showed low bias concerns since all studies used MRI and AI as an index text and histopathology as a reference standard, respectively.

## Discussion

The present study aimed to evaluate the diagnostic performance of various AI methods in glioma grading using MRI data. The findings highlighted the potential role of AI in differentiating glioma grades but also revealed variability in the models’ diagnostic performance, heterogeneity in the methods employed, and potential biases. Therefore, these results should be interpreted with caution.

The findings revealed that, among the evaluated AI methods, CNN models achieved the highest performance in glioma grading, with a sensitivity of 93% (95% CI: 88%–97%) and specificity of 92% (95% CI: 90%–94%). RF and SVM models, while slightly lower than CNN models, demonstrated comparable performance, with sensitivity and specificity of approximately 87%. Similarly, a previous study reported that CNN models significantly outperformed traditional ML approaches, such as SVM and RF, in glioma-grade prediction.^48^ In contrast, the analysis found that LR exhibited the lowest performance, with a sensitivity of 80% (95% CI: 70%–87%) and specificity of 79% (95% CI: 74%–83%). This result is not surprising, as LR is a relatively simple model constrained by its linear assumptions, making it less effective at capturing the complexity often inherent in medical imaging data.

The observed heterogeneity in sensitivity and specificity among methods reflects variations across the included studies. These differences may stem from the diversity of MRI techniques used, the selection of regions of interest (ROI), and the methods employed for feature extraction or reduction. The variability in MRI techniques among the included studies undoubtedly contributed to the observed heterogeneity. Several studies utilized conventional anatomical MRI images (T1-WI, T1-CE, T2-WI, FLAIR).^30,36,44^ One study relied solely on a single MRI method (CE-T1WI)^26^ and suggested that texture features derived from conventional anatomical MRI, particularly CE-T1WI, could yield more accurate classification results compared to other methods. Conversely, some studies employed a combination of conventional and advanced MRI techniques, including perfusion, diffusion, and MR spectroscopy.^32,40,42^ Two studies included in this meta-analysis relied on MR spectroscopy and reported that it could significantly enhance the noninvasive classification of brain tumors, particularly gliomas.^38,43^

Determining the volume of interest (VOl) in three-dimensional (3D) or the region of interest (ROI) in two-dimensional (2D) and defining the area to be calculated is a crucial first step in the AI pipeline. Segmenting images may be performed by a variety of methods automatically (using algorithms),^21,24^ semi-automatically,^28,32,36^ or manually.^40,42,44^ The different techniques used for VOIs/ROIs selection among the included studies could be one of the reasons for the high heterogeneity in our results.

Furthermore, the different feature extraction methods employed in the involved studies could be a subsidiary cause of the high heterogeneity in this result. Feature extraction has several methods and techniques for calculating gray-level characteristics, such as histogram, gray-level cooccurrence matrix (GLCM), gray-level size zone matrix (GLSZM), and intensity size zone (ISZ). Histogram parameters were the most used extracted feature among the included studies.^30,40,42^ GLCM features were the second most commonly used method after the histogram.^21,36,42^ Wavelet^28,37,39^ and gray-level size zone matrix (GLSZM)^22,32,39^ were also included in some studies. The least used features were based on ISZ, with only one study.^7^

Another possible contributor to the high heterogeneity in the results is the variety of feature reduction methods employed. The feature reduction or dimension reduction step is critical for generating valid and generalizable results, as it involves reducing the number of features to build statistical and machine learning models. This multi-step process excludes non-reproducible, redundant, and irrelevant features to improve model performance and reliability. Various feature reduction methods were used across the included studies. One such method was SVM-Lasso (least absolute shrinkage and selection operator), which has been reported to achieve higher accuracy than other assessed methods for feature selection. One study^25^ compared three different feature reduction techniques—PCA, RFE, and Lasso (principal component analysis, recursive feature elimination, and least absolute shrinkage and selection operator, respectively). The findings indicated that Lasso based on SVM had a slightly higher accuracy. SVM-RFE was utilized for feature reduction in other included studies.^26,40,42^ Additionally, the mRMR (minimum redundancy maximum relevance) method was employed in some studies.^7,43^ These variations in feature reduction techniques likely contributed to the observed heterogeneity in diagnostic performance among the studies.

Another potential reason for the higher heterogeneity index in the results is that some studies corrected the unbalanced sample size, while others^21,30,44^ did not. Several studies used SMOTE (Synthetic Minority Oversampling Technique) to statistically address data imbalance and mitigate bias.^22,40^ One study^22^ demonstrated that applying the SMOTE algorithm improved the handling of unbalanced data, as the classification of the high-dimensional, unbalanced datasets tends to be biased toward the majority class.

The statistically significant Egger test results for RF (*P* = .008) and CNN (*P* = .009) suggest a potential overrepresentation of studies reporting high-performing models. While this should not be interpreted as publication bias in the traditional sense used in hypothesis-driven research, it may reflect a broader trend in the literature toward selective reporting of successful results. Such a pattern can lead to an inflated perception of model performance in meta-analyses, particularly when less successful or non-optimized models are underreported. To mitigate this effect and improve the transparency and reliability of future syntheses, we encourage practices such as pre-registration of analysis protocols and comprehensive reporting of model performance, regardless of outcome.^49^

The high sensitivity and specificity demonstrated by CNNs and other AI models highlight their potential to assist clinicians in glioma grading, particularly in resource-limited settings where access to expert neuropathologists or advanced imaging technologies may be restricted. Integrating AI tools into the diagnostic workflow can enable healthcare providers to achieve more accurate and consistent grading, which is essential for effective treatment planning and prognostication. Future research should focus on multi-center validation studies involving large, standardized, and diverse patient cohorts to improve the generalizability and reliability of these AI models. Such efforts will be critical for establishing the robustness of AI applications in real-world clinical settings.

Several limitations were identified in this study. A key limitation of this review is the temporal mismatch between the classification systems used in the included studies and the current WHO guidelines. The most recent 2021-WHO classification^50^ emphasizes molecular diagnostics and redefines glioma categories, classifying them into grades 2, 3, or 4 based on both morphological and molecular features. However, the studies reviewed were conducted before or during the early adoption of this updated system. As a result, most relied on the WHO 2007 or 2016 classifications, which were the standard at the time. Consequently, the AI models analyzed were trained and validated using the WHO-2016/2007 glioma grading criteria. This discrepancy may limit the relevance of the findings to current clinical practice, where molecular markers are now central to diagnosis and prognosis under the WHO-2021 framework. Future research should focus on evaluating AI algorithms using datasets labeled according to the WHO-2021 criteria to ensure clinical applicability. In addition, several methodological constraints were also identified. First, the results are highly heterogeneous, which is expected given the factors mentioned earlier, as well as the variability in MRI measurements across centers and even between platforms within the same center. However, this heterogeneity was addressed during the analysis stage through the use of a random-effects model. Second, publication bias was detected for both the RF and CNN models, potentially due to the limited number of studies included for these models. Third, the small datasets in this meta-analysis constrain the statistical power of the findings. Nonetheless, this limitation underscores the adherence to strict methodological standards to remain faithful to the research question.

While this meta-analysis aggregates reported performance across studies, future research should aim to perform direct, head-to-head evaluations of models on standardized public datasets such as BraTS and TCIA. Such empirical benchmarking, particularly using publicly released code and independent test sets, would provide a more robust basis for model comparison and generalizability assessment.

## Conclusion

This study highlights the significant potential of AI-based methods, particularly CNNs, in glioma grading. CNNs demonstrated superior diagnostic performance compared to other AI models, highlighting their promise in enhancing accuracy and efficiency in clinical workflows. However, the integration of these tools into routine clinical practice necessitates rigorous validation to ensure reliability, standardization of methodologies to reduce variability, and effective mitigation of biases to promote equitable application. Addressing these challenges is crucial to fully realizing the transformative potential of AI in glioma management. With careful implementation and ongoing refinement, AI has the capacity to revolutionize diagnostic pathways, enabling more precise and timely interventions. This, in turn, could significantly improve outcomes for glioma patients on a global scale, ultimately advancing the field of neuro-oncology.

## Supplementary Material

vdaf162_suppl_Supplementary_Material
