# Supplementary material for: Assessment of the emerging role of AI in diagnosing gliomas using MRI: Systematic review and meta-analysis
Source: Neurooncol Adv. 2025 Aug 5;7(1):vdaf162. doi: 10.1093/noajnl/vdaf162 (PMC12448723; doi:10.1093/noajnl/vdaf162)
Supplement: vdaf162_suppl_Supplementary_Material [file vdaf162_suppl_supplementary_material.docx]

| Table 1A: Shows how outcomes from the same study were treated as originating from separate studies and the diagnostic performance of each | | | | |  |
| --- | --- | --- | --- | --- | --- |
| NO. | Author (years) | AI methods | Sensitivity | Specificity | |
| 1 | (Zöllner, Emblem and Schad, 2012), PCC^c^ | SVM | 0.89 | 0.77 | |
| 1 | (Zöllner, Emblem and Schad, 2012), PCA^c^ | SVM | 0.87 | 0.84 | |
| 1 | (Zöllner, Emblem and Schad, 2012), ICA^c^ | SVM | 0.87 | 0.75 | |
| 2 | (Wu *et al.*, 2015), SIC^b^ | SVM | 0.82 | 0.96 | |
| 2 | (Wu *et al.*, 2015), fALFF^b^ | SVM | 0.60 | 0.74 | |
| 2 | (Wu *et al.*, 2015), ReHo^b^ | SVM | 0.58 | 0.51 | |
| 2 | (Wu *et al.*, 2015), SIC^c^ and fALFF^b^ | SVM | 0.79 | 0.94 | |
| 2 | (Wu *et al.*, 2015), SIC^c^ and ReHo^b^ | SVM | 0.85 | 0.85 | |
| 2 | (Wu *et al.*, 2015), fALFF^b^ and ReHo^b^ | SVM | 0.64 | 0.57 | |
| 2 | (Wu *et al.*, 2015), SIC^c^ and fALFF^b^ and ReHo^b^ | SVM | 0.81 | 0.87 | |
| 3 | (Ranjith *et al.*, 2015) | SVM | 0.833 | 0.755 | |
| 4 | (Cheng *et al.*, 2022), NET^d^ | SVM | 0.911 | 0.817 | |
| 4 | (Cheng *et al.*, 2022), TC^d^ | SVM | 0.890 | 0.792 | |
| 5 | (Tian *et al.*, 2018), Overall^a^, Texture^b^ | SVM | 0.964 | 0.973 | |
| 5 | (Tian *et al.*, 2018), Diffusion^a^, Texture^b^ | SVM | 0.919 | 0.946 | |
| 5 | (Tian *et al.*, 2018), Structure^a^, Texture^b^ | SVM | 0.919 | 0.937 | |
| 5 | (Tian *et al.*, 2018), Perfusion^a^, Texture^b^ | SVM | 0.892 | 0.865 | |
| 5 | (Tian *et al.*, 2018), Overall^a^, histogram^b^ | SVM | 0.838 | 0.991 | |
| 5 | (Tian *et al.*, 2018), Diffusion^a^, histogram^b^ | SVM | 0.847 | 0.991 | |
| 5 | (Tian *et al.*, 2018), Structure^a^, histogram^b^ | SVM | 0.874 | 0.937 | |
| 5 | (Tian *et al.*, 2018), Perfusion^a^, histogram^b^ | SVM | 0.892 | 0.847 | |
| 5 | (Tian *et al.*, 2018), Overall^a^, mean^b^ | SVM | 0.838 | 0.955 | |
| 5 | (Tian *et al.*, 2018), Diffusion^a^, mean^b^ | SVM | 0.82 | 0.901 | |
| 5 | (Tian *et al.*, 2018), Structure^a^, mean^b^ | SVM | 0.703 | 0.946 | |
| 5 | (Tian *et al.*, 2018), Perfusion^a^, mean^b^ | SVM | 0.802 | 0.784 | |
| 6 | (Bisdas *et al.*, 2018), difusional kurtosis imaging (DKI)^a^ | SVM | 0.79 | 0.70 | |
| 6 | (Bisdas *et al.*, 2018), conventional (FLAIR)^a^ | SVM | 0.79 | 0.54 | |
| 6 | (Bisdas *et al.*, 2018), both difusional kurtosis imaging (DKI)^a^ and conventional (FLAIR)^a^ | SVM | 0.63 | 0.31 | |
| 7 | (Cho *et al.*, 2018) | SVM | 0.9714 | 0.6933 | |
| 8 | (Vamvakas *et al.*, 2019) | SVM | 0.95 | 0.96 | |
| 9 | (Qi *et al.*, 2019) | SVM | 0.742 | 0.814 | |
| 10 | (Zhang *et al.*, 2020) | SVM | 0.98 | 0.86 | |
| 11 | (Hashido, Saito and Ishida, 2021), Original sample size, (RFE)^c^ SVM-RBF as a classifier^g^ | SVM | 0.917 | 0.917 | |
| 11 | (Hashido, Saito and Ishida, 2021), Original sample size, (RFE)^c^ SVM-L as a classifier^g^ | SVM | 1 | 0.833 | |
| 11 | (Hashido, Saito and Ishida, 2021), SMOTE^e^ sample size, (RFE)^c^ SVM-RBF as a classifier^g^ | SVM | 0.875 | 0.917 | |
| 11 | (Hashido, Saito and Ishida, 2021), SMOTE^e^ sample size, (RFE)^c^ SVM-L as a classifier^g^ | SVM | 0.875 | 0.875 | |
| 12 | (Malik *et al.*, 2021), ANOVA F-test^c^ | SVM | 0.714 | 0.694 | |
| 12 | (Malik *et al.*, 2021), (mRMR)^c^ | SVM | 0.857 | 0.806 | |
| 12 | (Malik *et al.*, 2021), (RFE)^c^ | SVM | 0.762 | 0.861 | |
| 13 | (Chen *et al.*, 2021) | SVM | 0.81 | 0.67 | |
| 14 | (Jiang *et al.*, 2022), SVM-PCA^c^ | SVM | 0.754 | 0.890 | |
| 14 | (Jiang *et al.*, 2022), SVM-RFE^c^ | SVM | 0.869 | 0.800 | |
| 14 | (Jiang *et al.*, 2022), SVM-LASSO^c^ | SVM | 0.771 | 0.920 | |
| 15 | (Ding *et al.*, 2022) | SVM | 0.741 | 0.907 | |
| 4 | (Cheng *et al.*, 2022), conventional^a^, NET^d^ | LR | 0.902 | 0.885 | |
| 4 | (Cheng *et al.*, 2022), conventional^a^, TC^d^ | LR | 0.881 | 0.801 | |
| 7 | (Cho *et al.*, 2018), conventional^a^ | LR | 0.9643 | 0.6800 | |
| 16 | (Ditmer *et al.*, 2018), T1-CE^a^ | LR | 0.93 | 0.86 | |
| 16 | (Ditmer *et al.*, 2018), FLAIR^a^ | LR | 0.81 | 0.65 | |
| 16 | (Ditmer *et al.*, 2018), DWI^a^ | LR | 0.85 | 0.81 | |
| 17 | (Su *et al.*, 2021), ADC^a^ | LR | 0.791 | 0.75 | |
| 17 | (Su *et al.*, 2021), Dmean^a^ | LR | 0.804 | 0.733 | |
| 17 | (Su *et al.*, 2021), FA^a^ | LR | 0.63 | 0.66 | |
| 17 | (Su *et al.*, 2021), T2 FLAIR^a^ | LR | 0.703 | 0.736 | |
| 17 | (Su *et al.*, 2021), MK^a^ | LR | 0.738 | 0.67 | |
| 17 | (Su *et al.*, 2021), all^a^ | LR | 0.762 | 0.747 | |
| 11 | (Hashido, Saito and Ishida, 2021), conventional and advanced MRI, Original sample | LR | 1 | 0.667 | |
| 11 | (Hashido, Saito and Ishida, 2021), conventional and advanced MRI, SMOTE^e^ sample | LR | 0.917 | 0.958 | |
| 18 | (Huang *et al.*, 2021), T1-CE^a^, radiomics^b^ features | LR | 0.9130 | 0.8460 | |
| 18 | (Huang *et al.*, 2021), T2-W^a^, radiomics^b^ features | LR | 0.5870 | 0.9230 | |
| 18 | (Huang *et al.*, 2021), T1-W^a^, radiomics^b^ features | LR | 0.8040 | 0.6150 | |
| 18 | (Huang *et al.*, 2021), T1-CE^a^, radiomics^b^ features and Clinical features | LR | 0.9350 | 0.8460 | |
| 18 | (Huang *et al.*, 2021), T2-W^a^, radiomics^b^ features and Clinical features | LR | 0.6990 | 0.9230 | |
| 18 | (Huang *et al.*, 2021), T 1-W^a^, radiomics^b^ features and Clinical features | LR | 0.848 | 0.6920 | |
| 19 | (Zhou *et al.*, 2022), conventional^a^ | LR | 1.0 | 0.778 | |
| 15 | (Ding *et al.*, 2022), Radiomics^b^ + VGG16^b^ | LR | 0.810 | 0.907 | |
| 20 | (Ge *et al.*, 2020), T1\T1ce\T2\FLAIR^a^ | CNN | 0.7826 | 0.9365 | |
| 21 | (Özcan *et al.*, 2021), (AlexNet)^f^, T2W\FLAIR^a^ | CNN | 0.940 | 0.907 | |
| 21 | (Özcan *et al.*, 2021), (GoogleNet)^f^, T2W\FLAIR^a^ | CNN | 0.980 | 0.889 | |
| 21 | (Özcan *et al.*, 2021), (SqueezeNet)^f^, T2W\FLAIR^a^ | CNN | 0.920 | 0.870 | |
| 21 | (Özcan *et al.*, 2021), (Custom)^f^, T2W\FLAIR^a^ | CNN | 0.980 | 0.963 | |
| 22 | (He *et al.*, 2021), Four modalities (T1, T1ce, T2, and T2-FLAIR)^a^ | CNN | 0.9333 | 0.92 | |
| 22 | (He *et al.*, 2021), Two modalities (T1ce, T2-FLAIR)^a^ | CNN | 0.9524 | 0.92 | |
| 3 | (Ranjith *et al.*, 2015) | RF | 0.806 | 0.857 | |
| 4 | (Cheng *et al.*, 2022), NET^d^ | RF | 0.958 | 0.856 | |
| 4 | (Cheng *et al.*, 2022), TC^d^ | RF | 0.924 | 0.805 | |
| 7 | (Cho *et al.*, 2018) | RF | 1 | 1 | |
| 23 | (De Looze *et al.*, 2018) | RF | 0.82 | 0.94 | |
| 24 | (Zhao *et al.*, 2020), Radiomics^b^ (T1_CE)^a^ | RF | 0.672 | 0.789 | |
| 24 | (Zhao *et al.*, 2020), Radiomics^b^ (FLAIR)^a^ | RF | 0.700 | 0.683 | |
| 24 | (Zhao *et al.*, 2020), Radiomics^b^ (T1 CE + FLAIR)^a^ | RF | 0.778 | 0.783 | |
| 24 | (Zhao *et al.*, 2020), neuroradiologist 1 | RF | 0.824 | 0.632 | |
| 24 | (Zhao *et al.*, 2020), neuroradiologist 2 | RF | 0.706 | 0.684 | |
| 24 | (Zhao *et al.*, 2020), neuroradiologist 3 | RF | 0.647 | 0.632 | |
| 25 | (Gao *et al.*, 2020) | RF | 0.63 | 0.89 | |
| 11 | (Hashido, Saito and Ishida, 2021), Original sample size | RF | 1 | 1 | |
| 11 | (Hashido, Saito and Ishida, 2021), SMOTE^e^ sample size | RF | 1 | 1 | |
| 15 | (Ding *et al.*, 2022) | RF | 0.793 | 0.837 | |

*** Note:** ^a^MRI methods, ^b^Feature Extraction Method, ^c^Feature Reduction Method, ^d^VOIs, ^e^Sample Balancing method, ^f^transfer learning, ^g^AI classifier type. Abbreviations: PCA: Principal component analysis, PPC: Pearson’s correlation coefficients, RBF: Radial basis function, ReHo: Regional Homogeneity, RFE: Recessive feature extraction, SIC: Signal intensity correlation, SMOTE: Synthetic Minority Oversampling Technique, SVM: Support vector machine, SVM-L: SVM with the linear kernel, TC: tumor core, T1-CE: T1 weighted image with contrast, T1-W: T1 weighted image, T2-W: T2 weighted image. VGG: Visual Geometry Group, ADC: Apparent diffusion coefficient, ANOVA: Analysis of Variance, FA: Fractional anisotropy, DKI: Diffusion kurtosis imaging, Dmean: Mean diffusion coefficient, DWI: Diffusion-weighted imaging, fALFF: Fractional amplitude of low-frequency fluctuations, FLAIR: Fluid-attenuated inversion recovery, ICA: Independent component analysis, LASSO: Least absolute shrinkage and selection operator, MK: Mean kurtosis, mRMR: Maximum Relevance — Minimum Redundancy, NET: non-enhancing tumor

| Table 2A Meta-Regression Summary | | | | | | |
| --- | --- | --- | --- | --- | --- | --- |
| AI model | Moderator | R2 | QM | QE | τ² | I² (%) |
| SVM | Features extraction | 50% | QM(df = 15) = 36.043, p-val = 0.002 | QE(df = 28) = 85.113, p-val < .001 | 0.79 | 71.89% |
|  | Feature reduction | 0% | QM(df = 12) = 11.820, p-val = 0.460 | QE(df = 31) = 131.769, p-val < .001 | 1.67 | 82.46% |
|  | ROI selection | 56% | QM(df = 13) = 44.230, p-val < .001 | QE(df = 30) = 83.742, p-val < .001 | 0.68 | 66% |
|  | MRI image weight | 80.54% | QM(df = 17) = 82.309, p-val < .001 | QE(df = 26) = 48.589, p-val = 0.005 | 0.31 | 47% |
|  | Class imbalance correction | 40% | QM(df = 1) = 17.111, p-val < .001 | QE(df = 42) = 136.636, p-val < .001 | 0.91 | 74% |
| LR | Features extraction | 0% | QM(df = 2) = 1.309, p-val = 0.520 | QE(df = 19) = 87.268, p-val < .001 | 1.77 | 86% |
|  | Feature reduction | 61% | QM(df = 6) = 19.839, p-val = 0.003 | QE(df = 15) = 45.335, p-val < .001 | 0.67 | 72% |
|  | ROI selection | 60% | QM(df = 7) = 21.393, p-val = 0.003 | QE(df = 14) = 39.366, p-val < .001 | 0.69 | 69% |
|  | MRI image weight | 63% | QM(df = 12) = 28.946, p-val = 0.004 | QE(df = 9) = 24.126, p-val = 0.004 | 0.64 | 64% |
|  | Class imbalance correction | 21% | QM(df = 1) = 4.656, p-val = 0.031 | QE(df = 20) = 79.112, p-val < .001 | 1.36 | 82% |
| RF | Features extraction | 0% | QM(df = 4) = 4.912, p-val = 0.296 | QE(df = 10) = 65.922, p-val < .001 | 2.67 | 89% |
|  | Feature reduction | 100% | QM(df = 6) = 72.543, p-val < .001 | QE(df = 8) = 7.079, p-val = 0.528 | 0 | 0% |
|  | ROI selection | 100% | QM(df = 7) = 77.082, p-val < .001 | QE(df = 7) = 2.539, p-val = 0.924 | 0 | 0% |
|  | MRI image weight | 90.7% | QM(df = 7) = 37.274, p-val < .001 | QE(df = 7) = 17.524, p-val = 0.014 | 0.23 | 39.71% |
|  | Class imbalance correction | 40.79% | QM(df = 1) = 3.157, p-val = 0.076 | QE(df = 13) = 59.453, p-val < .001 | 1.46 | 81.53% |
| Moderators: study-level variable to explain heterogeneity in the effect sizes across studies.  R²: Shows how much heterogeneity the moderator explains.  QM: Impact of the moderator on the outcome heterogeneity  QE: How much heterogeneity remains after accounting for the moderator.  τ² and I²: Quantify residual heterogeneity and help compare models (e.g., before vs after adding moderators). τ²: Residual between-study variance after accounting for moderators. I² (%): Percentage of residual heterogeneity (not explained). | | | | | | |


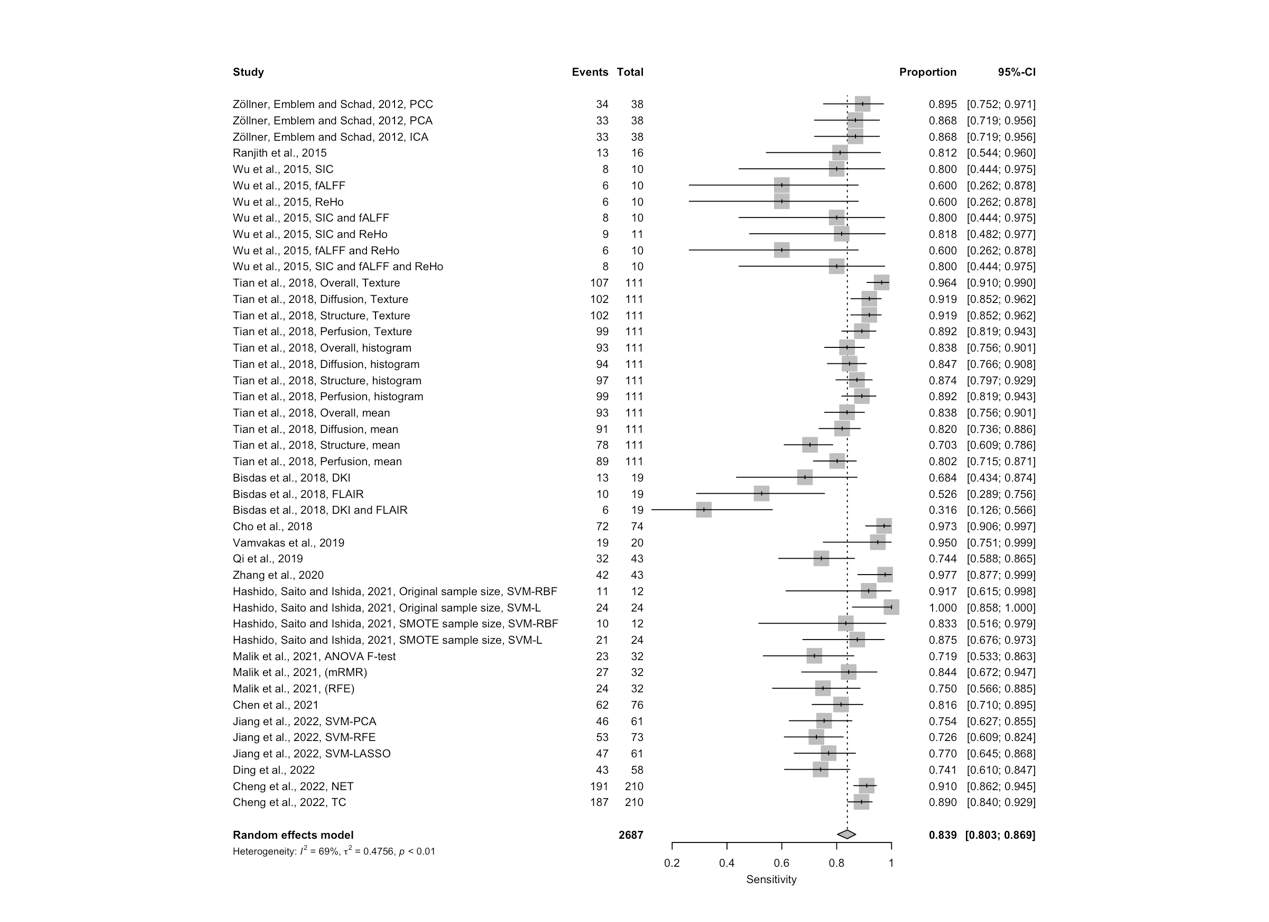


Figure 1A Forest plot of the pooled sensitivity from SVM, the dotted vertical line represents the pooled effect size point where the effect size in individual studies has a very different distribution (heterogeneity) around this line. The diamond at the bottom represents the pooled effect and its 95% CI. CI, confidence interval.


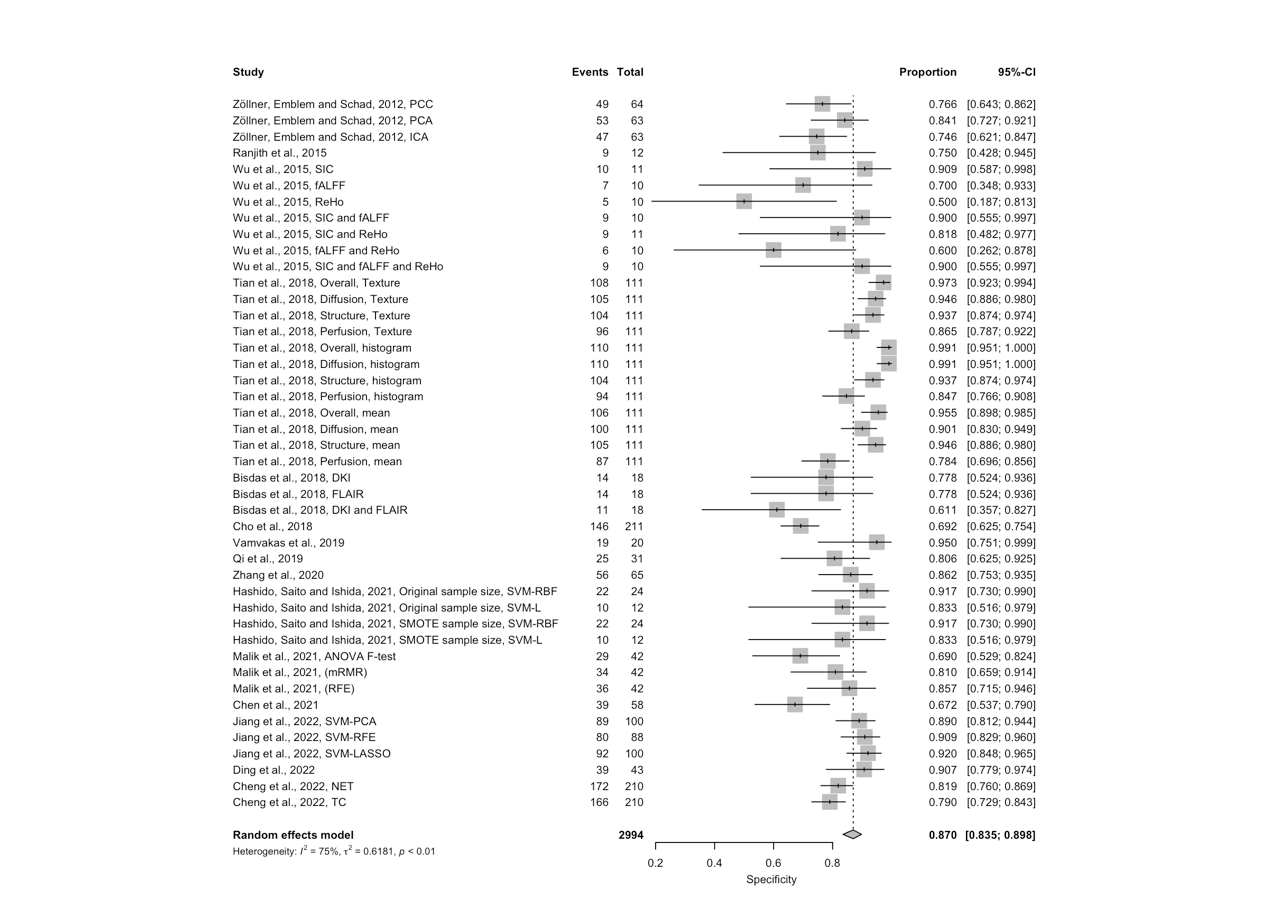


Figure 2A Forest plot of the pooled specificity from SVM, the dotted vertical line represents the pooled effect size point where the effect size in individual studies has a very different distribution (heterogeneity) around this line. The diamond at the bottom represents the pooled effect and its 95% CI. CI, confidence interval.


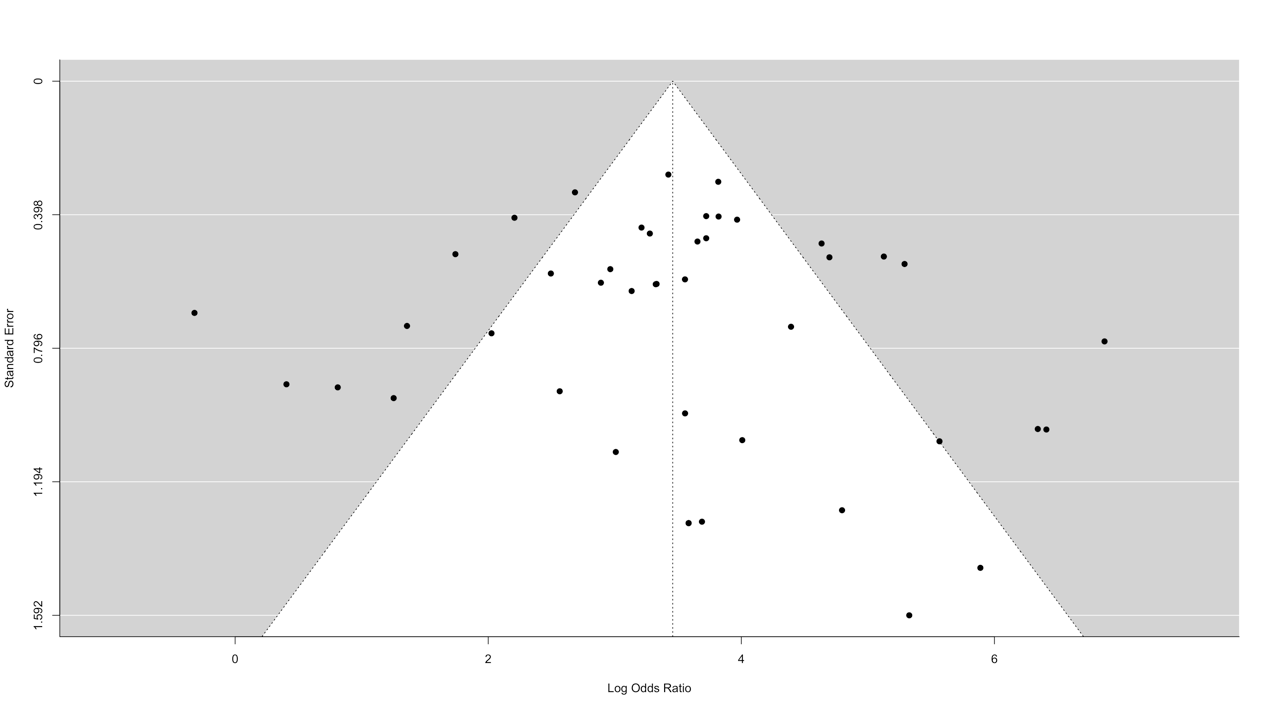


Figure 3A The funnel plot of SVM is symmetric and does not show publication bias.


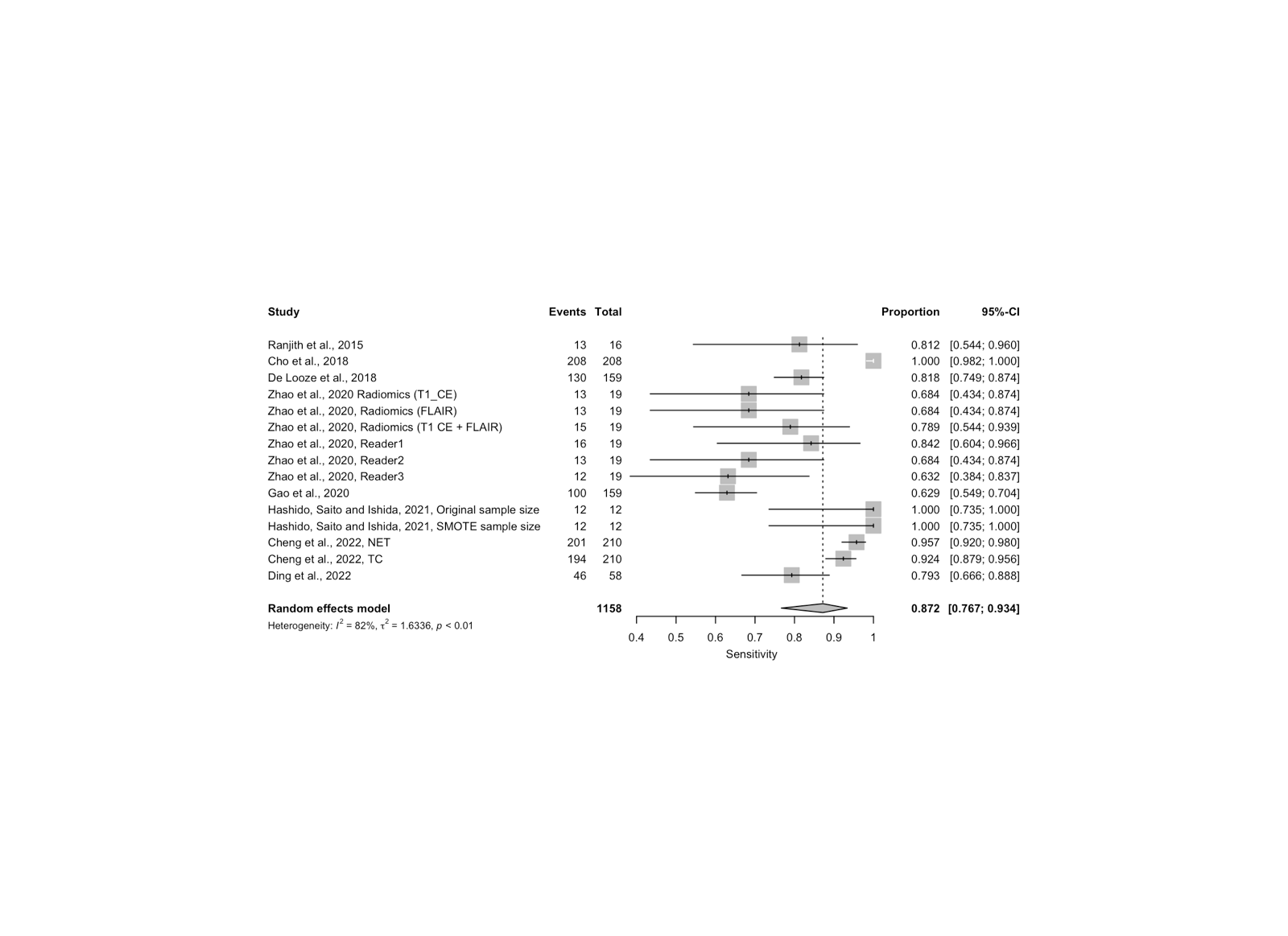


Figure 4A Forest plot of the pooled sensitivity from RF, the dotted vertical line represents the pooled effect size point where the effect size in individual studies has a very different distribution (heterogeneity) around this line. The diamond at the bottom represents the pooled effect and its 95% CI. CI, confidence interval.


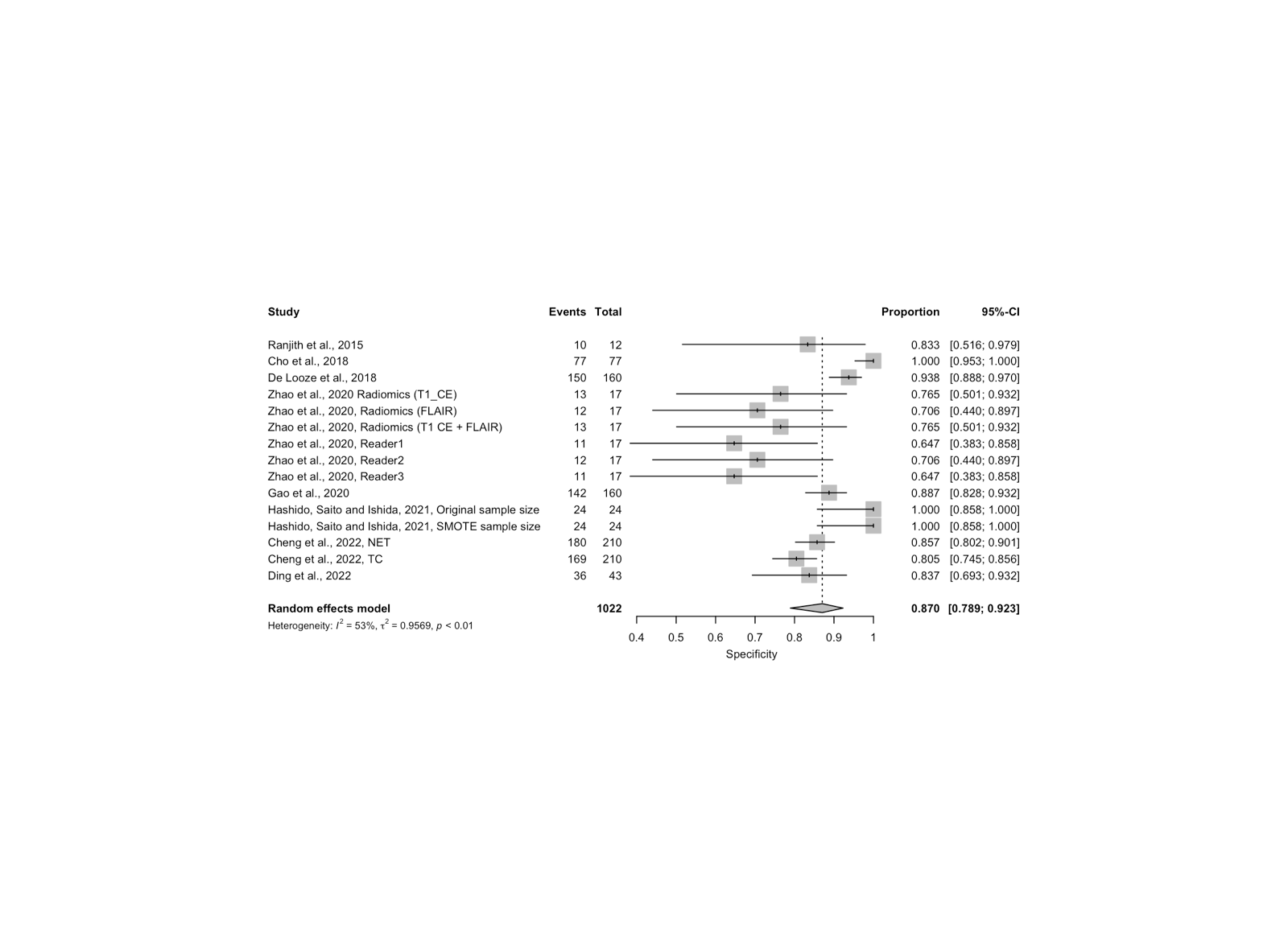


Figure 5A Forest plot of the pooled specificity from RF, the dotted vertical line represents the pooled effect size point where the effect size in individual studies has a very different distribution (heterogeneity) around this line. The diamond at the bottom represents the pooled effect and its 95% CI. CI, confidence interval.


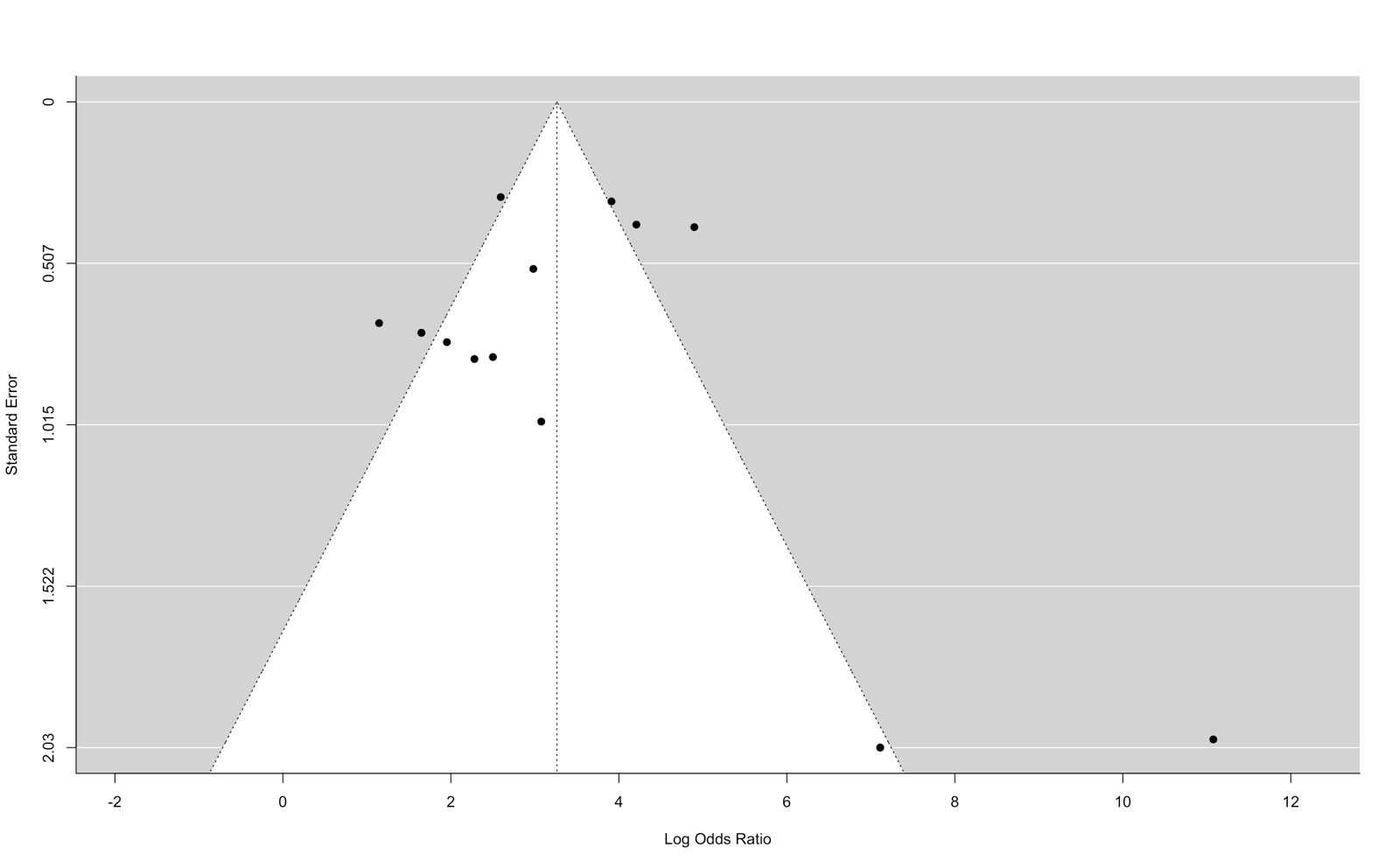


Figure 6A The funnel plot of RF is asymmetric and does not show publication bias.


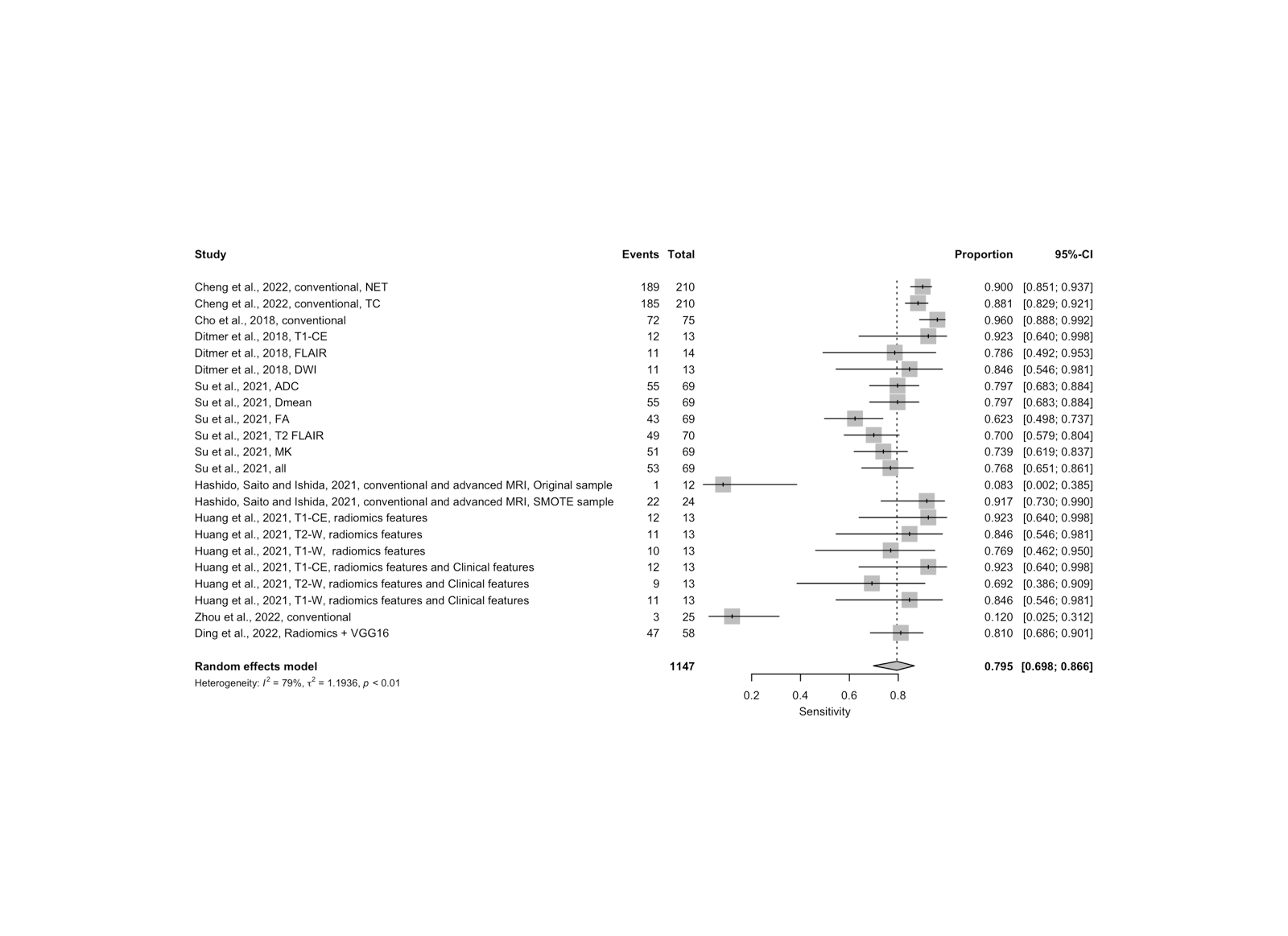


Figure 7A Forest plot of the pooled sensitivity from LR, the dotted vertical line represents the pooled effect size point where the effect size in individual studies has a very different distribution (heterogeneity) around this line. The diamond at the bottom represents the pooled effect and its 95% CI. CI, confidence interval.


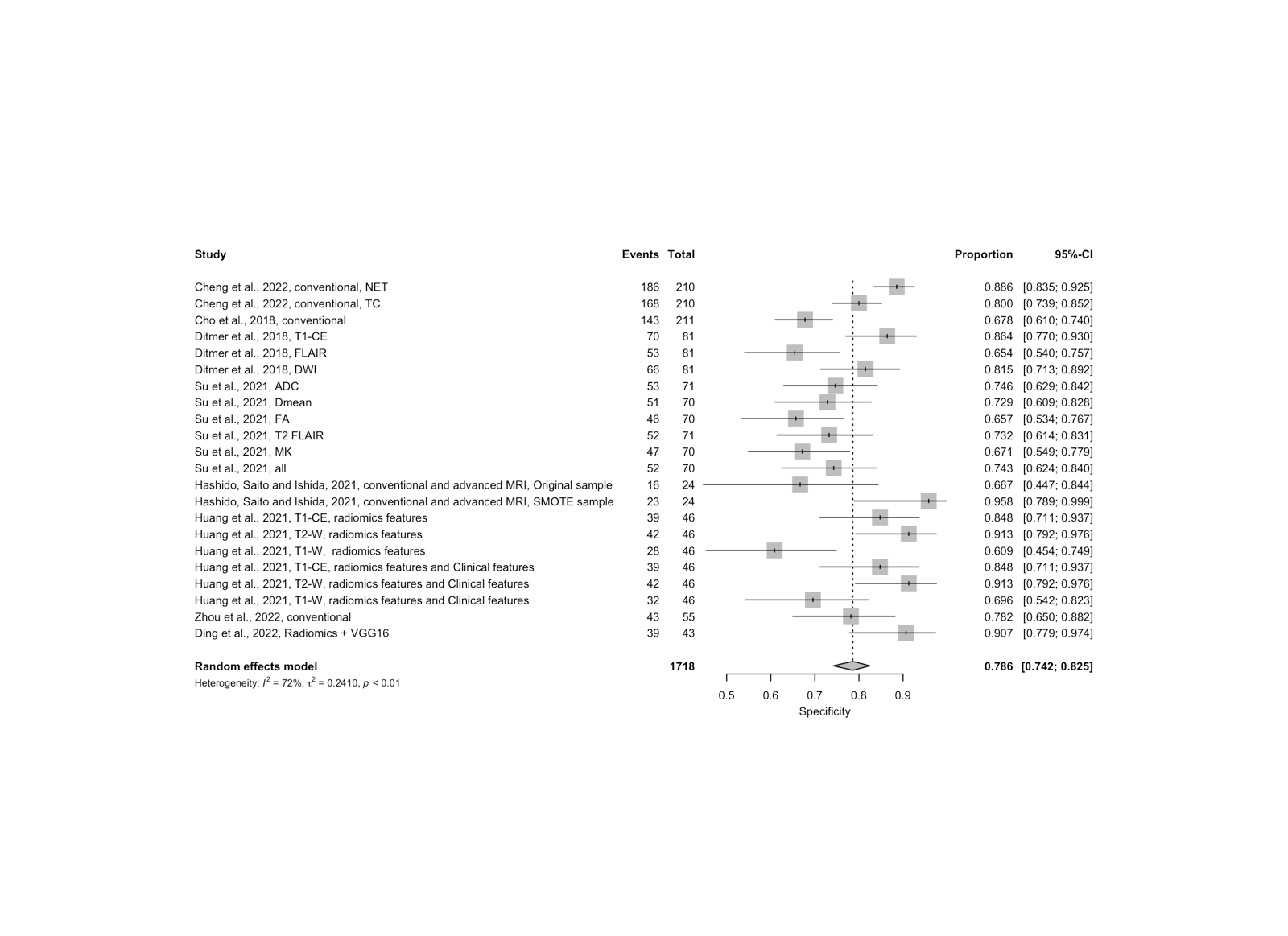


Figure 8A Forest plot of the pooled specificity from LR the dotted vertical line represents the pooled effect size point where the effect size in individual studies has a very different distribution (heterogeneity) around this line. The diamond at the bottom represents the pooled effect and its 95% CI. CI, confidence interval.


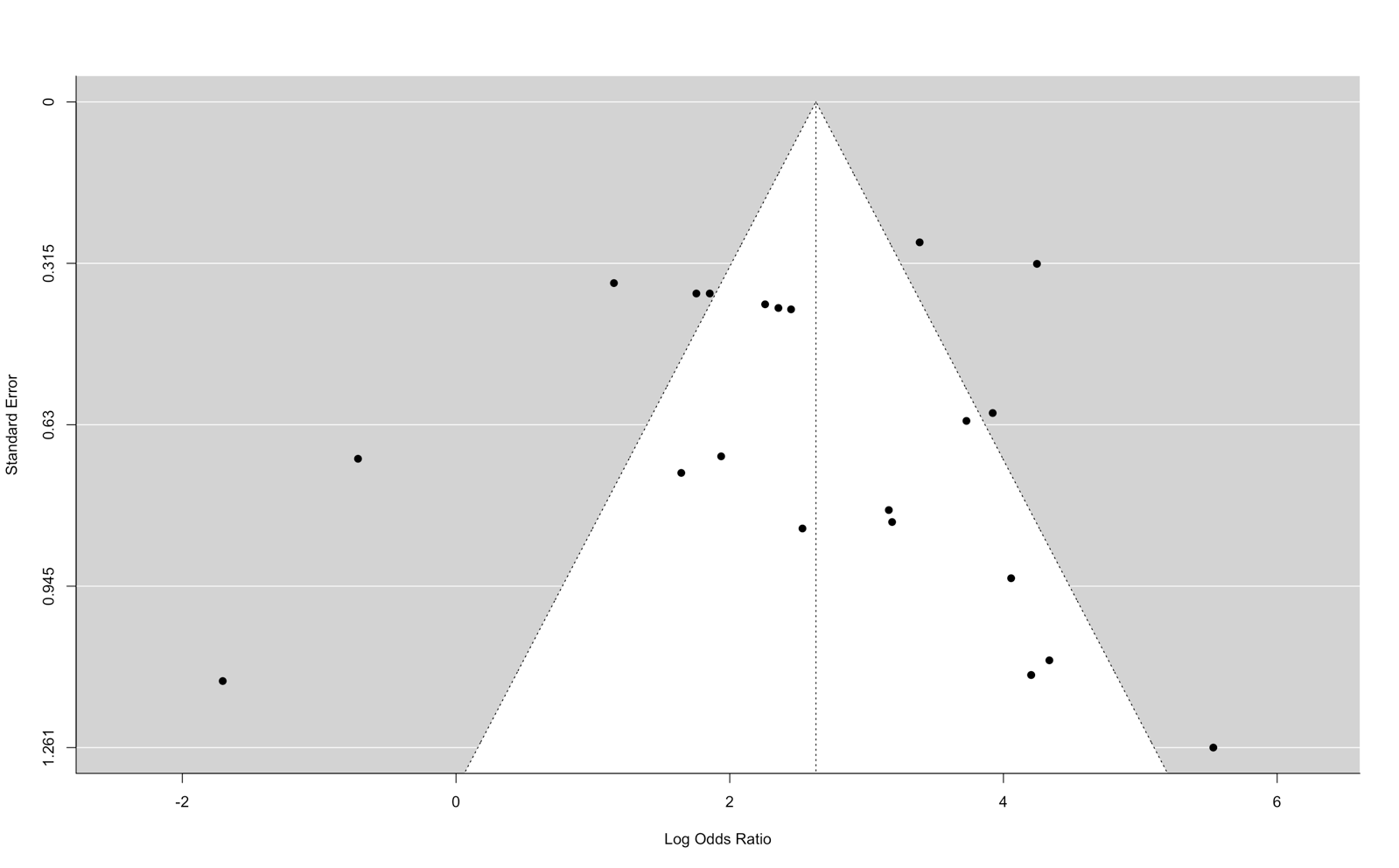


Figure 9A The funnel plot of LR is symmetric and does not show publication bias.


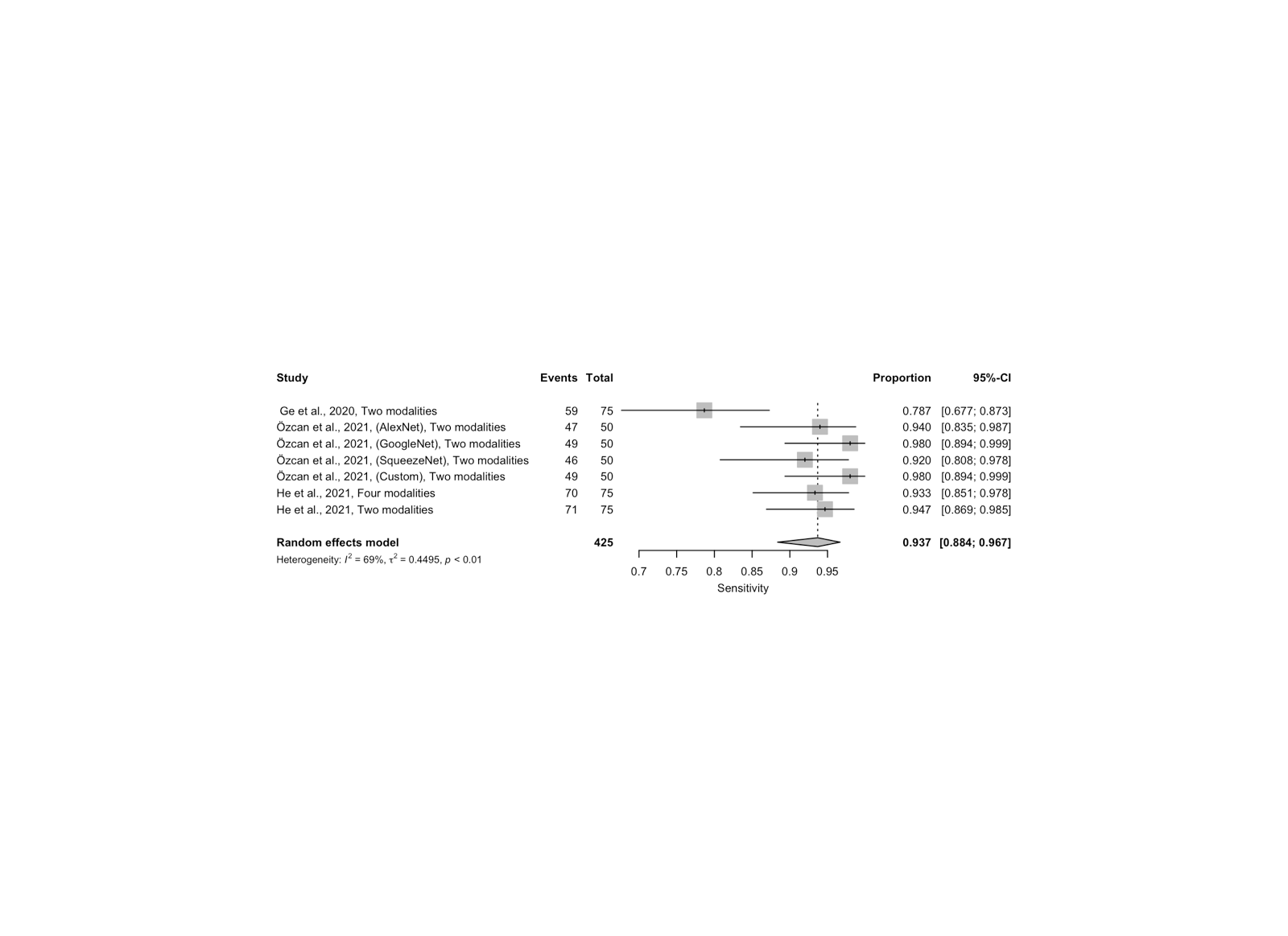


Figure 10A Forest plot of the pooled sensitivity from CNN, the dotted vertical line represents the pooled effect size point where the effect size in individual studies has a very different distribution (heterogeneity) around this line. The diamond at the bottom represents the pooled effect and its 95% CI. CI, confidence interval.


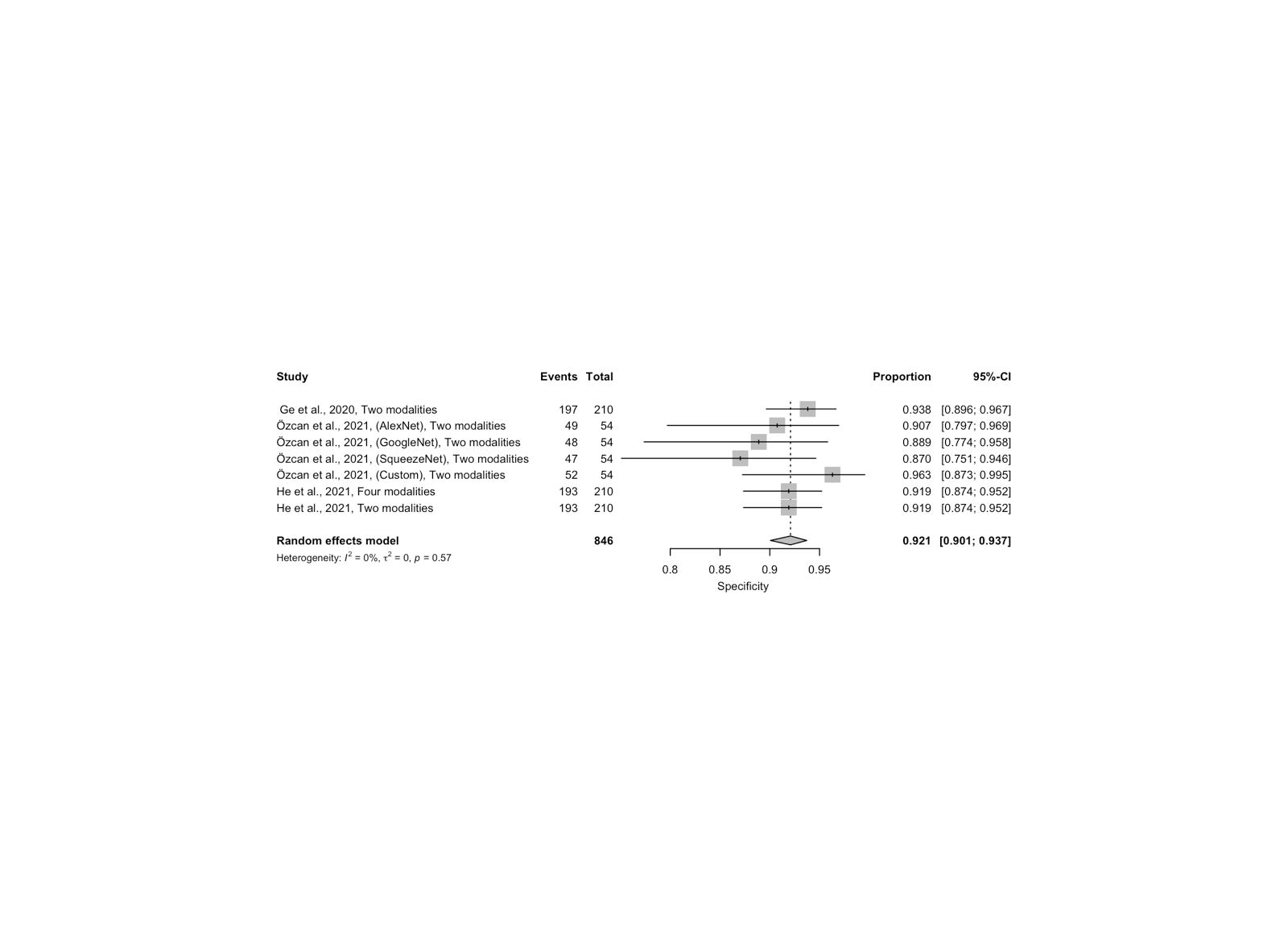


Figure 11A Forest plot of the pooled specificity from CNN the dotted vertical line represents the pooled effect size point where the effect size in individual studies has a very different distribution (heterogeneity) around this line. The diamond at the bottom represents the pooled effect and its 95% CI. CI, confidence interval.


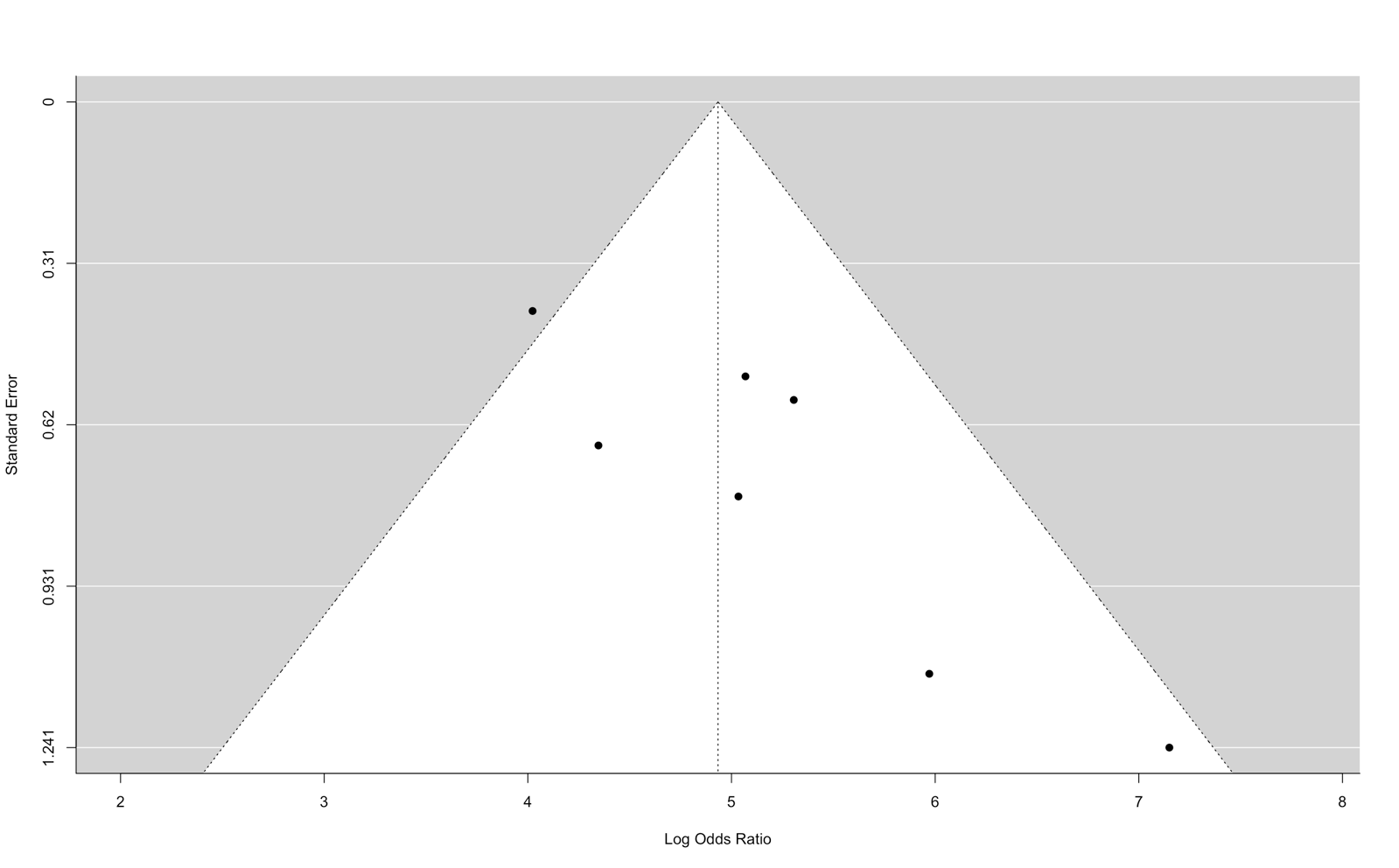


Figure 12A The funnel plot of CNN is asymmetric and does not show publication bias.

References:

Bisdas, S. *et al.* (2018) ‘Texture analysis- and support vector machine-assisted diffusional kurtosis imaging may allow in vivo gliomas grading and IDH-mutation status prediction: a preliminary study’, *Scientific Reports*. England, 8(1), p. 6108. doi: 10.1038/s41598-018-24438-4.

Chen, T. *et al.* (2021) ‘Detection and Grading of Gliomas Using a Novel Two-Phase Machine Learning Method Based on MRI Images.’, *Frontiers in neuroscience*. Switzerland, 15, p. 650629. doi: 10.3389/fnins.2021.650629.

Cheng, J. *et al.* (2022) ‘Prediction of Glioma Grade Using Intratumoral and Peritumoral Radiomic Features From Multiparametric MRI Images.’, *IEEE/ACM transactions on computational biology and bioinformatics*. United States, 19(2), pp. 1084–1095. doi: 10.1109/TCBB.2020.3033538.

Cho, H. ho *et al.* (2018) ‘Classification of the glioma grading using radiomics analysis’, *PeerJ*, 2018(11), pp. 1–17. doi: 10.7717/peerj.5982.

Ding, J. *et al.* (2022) ‘Developing and validating a deep learning and radiomic model for glioma grading using multiplanar reconstructed magnetic resonance contrast-enhanced T1-weighted imaging: a robust, multi-institutional study.’, *Quantitative imaging in medicine and surgery*. China, 12(2), pp. 1517–1528. doi: 10.21037/qims-21-722.

Ditmer, A. *et al.* (2018) ‘Diagnostic accuracy of MRI texture analysis for grading gliomas’, *Journal of Neuro-Oncology*. United States, 140(3), pp. 583–589. doi: 10.1007/s11060-018-2984-4.

Gao, M. *et al.* (2020) ‘Machine Learning-Based Radiomics Predicting Tumor Grades and Expression of Multiple Pathologic Biomarkers in Gliomas’, *Frontiers in Oncology*. Switzerland, 10, p. 1676. doi: 10.3389/fonc.2020.01676.

Ge, C. *et al.* (2020) ‘Deep semi-supervised learning for brain tumor classification’, *BMC Medical Imaging*, 20(1), p. 87. doi: 10.1186/s12880-020-00485-0.

Hashido, T., Saito, S. and Ishida, T. (2021) ‘Radiomics-Based Machine Learning Classification for Glioma Grading Using Diffusion- And Perfusion-Weighted Magnetic Resonance Imaging’, *Journal of Computer Assisted Tomography*, 45(4), pp. 606–613. doi: 10.1097/RCT.0000000000001180.

He, M. *et al.* (2021) ‘Hierarchical-order multimodal interaction fusion network for grading gliomas’, *Physics in Medicine and Biology*. IOP Publishing, 66(21), p. 215016. doi: 10.1088/1361-6560/ac30a1.

Huang, W.-Y. *et al.* (2021) ‘Comparison of Radiomics Analyses Based on Different Magnetic Resonance Imaging Sequences in Grading and Molecular Genomic Typing of Glioma.’, *Journal of computer assisted tomography*. United States, 45(1), pp. 110–120. doi: 10.1097/RCT.0000000000001114.

Jiang, L. *et al.* (2022) ‘Machine Learning Based on Diffusion Kurtosis Imaging Histogram Parameters for Glioma Grading.’, *Journal of clinical medicine*. Switzerland, 11(9). doi: 10.3390/jcm11092310.

De Looze, C. *et al.* (2018) ‘Machine learning: a useful radiological adjunct in determination of a newly diagnosed glioma’s grade and IDH status’, *Journal of Neuro-Oncology*, 139(2), pp. 491–499. doi: 10.1007/s11060-018-2895-4.

Malik, N. *et al.* (2021) ‘MRI radiomics to differentiate between low grade glioma and glioblastoma peritumoral region.’, *Journal of neuro-oncology*. United States, 155(2), pp. 181–191. doi: 10.1007/s11060-021-03866-9.

Özcan, H. *et al.* (2021) ‘A comparative study for glioma classification using deep convolutional neural networks.’, *Mathematical biosciences and engineering : MBE*. United States, 18(2), pp. 1550–1572. doi: 10.3934/mbe.2021080.

Qi, C. *et al.* (2019) ‘A quantitative SVM approach potentially improves the accuracy of magnetic resonance spectroscopy in the preoperative evaluation of the grades of diffuse gliomas.’, *NeuroImage. Clinical*. Netherlands, 23, p. 101835. doi: 10.1016/j.nicl.2019.101835.

Ranjith, G. *et al.* (2015) ‘Machine learning methods for the classification of gliomas: initial results using features extracted from MR spectroscopy’, *Neuroradiology Journal*, 28(2), pp. 106–111. doi: 10.1177/1971400915576637.

Su, C. *et al.* (2021) ‘T2-FLAIR, DWI and DKI radiomics satisfactorily predicts histological grade and Ki-67 proliferation index in gliomas’, *American Journal of Translational Research*, 13(8), pp. 9182–9194.

Tian, Q. *et al.* (2018) ‘Radiomics strategy for glioma grading using texture features from multiparametric MRI’, *Journal of Magnetic Resonance Imaging*, 48(6), pp. 1518–1528. doi: 10.1002/jmri.26010.

Vamvakas, A. *et al.* (2019) ‘Imaging biomarker analysis of advanced multiparametric MRI for glioma grading.’, *Physica medica : PM : an international journal devoted to the applications of physics to medicine and biology : official journal of the Italian Association of Biomedical Physics (AIFB)*. Italy, 60, pp. 188–198. doi: 10.1016/j.ejmp.2019.03.014.

Wu, J. *et al.* (2015) ‘Resting state fMRI feature-based cerebral glioma grading by support vector machine’, *International Journal of Computer Assisted Radiology and Surgery*. Germany, 10(7), pp. 1167–1174. doi: 10.1007/s11548-014-1111-z.

Zhang, Z. *et al.* (2020) ‘Deep Convolutional Radiomic Features on Diffusion Tensor Images for Classification of Glioma Grades.’, *Journal of digital imaging*. United States, 33(4), pp. 826–837. doi: 10.1007/s10278-020-00322-4.

Zhao, S.-S. *et al.* (2020) ‘Better efficacy in differentiating WHO grade II from III oligodendrogliomas with machine-learning than radiologist’s reading from conventional T1 contrast-enhanced and fluid attenuated inversion recovery images.’, *BMC neurology*. England, 20(1), p. 48. doi: 10.1186/s12883-020-1613-y.

Zhou, H. *et al.* (2022) ‘Application of Enhanced T1WI of MRI Radiomics in Glioma Grading’, *International Journal of Clinical Practice*, 2022. doi: 10.1155/2022/3252574.

Zöllner, F. G., Emblem, K. E. and Schad, L. R. (2012) ‘SVM-based glioma grading: Optimization by feature reduction analysis.’, *Zeitschrift fur medizinische Physik*. Germany, 22(3), pp. 205–214. doi: 10.1016/j.zemedi.2012.03.007.
